# Discovery of potent bisindole-based pyrazolopyridine derivatives as topoisomerase inhibitors: DNA damage induction and synergistic antileukemic activity

**DOI:** 10.3389/fphar.2026.1745220

**Published:** 2026-03-04

**Authors:** Wagdy M. Eldehna, Haytham O. Tawfik, Denisa Veselá, Miroslav Peřina, Ahmed T. Negmeldin, Zainab M. Elsayed, Taghreed A. Majrashi, Veronika Vojáčková, Mostafa M. Elbadawi, Moataz A. Shaldam, Vladimír Kryštof, Hatem A. Abdel-Aziz

**Affiliations:** 1 Department of Pharmaceutical Chemistry, Faculty of Pharmacy, Kafrelsheikh University, Kafrelsheikh, Egypt; 2 Department of Pharmaceutical Chemistry, Faculty of Pharmacy, Tanta University, Tanta, Egypt; 3 Department of Experimental Biology, Faculty of Science, Palacký University Olomouc, Olomouc, Czechia; 4 Department of Pharmaceutical Sciences, College of Pharmacy and Thumbay Research Institute for Precision Medicine, Gulf Medical University, Ajman, United Arab Emirates; 5 Department of Pharmaceutical Organic Chemistry, Faculty of Pharmacy, Cairo University, Cairo, Egypt; 6 Scientific Research and Innovation Support Unit, Faculty of Pharmacy, Kafrelsheikh University, Kafr El-Shaikh, Egypt; 7 Department of Pharmacognosy, College of Pharmacy, King Khalid University, Abha, Saudi Arabia; 8 Applied Organic Chemistry Department, National Research Center, Cairo, Egypt

**Keywords:** bis-indole, leukemia, molecular modeling, synergistic therapy, topoisomerase inhibitors

## Abstract

**Introduction:**

The development of novel anticancer agents targeting DNA replication and repair mechanisms remains a priority in leukemia therapy. In this study, newly synthesized derivatives incorporating bis-indole and pyrazolo[3,4-*b*]pyridine scaffolds were evaluated for their antiproliferative potential against leukemia cell lines.

**Methods:**

The antiproliferative activity of the synthesized compounds was assessed in four cancer cell lines, including acute myeloid leukemia (MV4-11) and chronic myeloid leukemia (K562). Growth inhibition (GI_50_) values were determined. DNA relaxation assays were performed to evaluate inhibition of topoisomerase I and IIα activities. Cell cycle distribution, apoptosis induction, and DNA damage response markers were analyzed using cellular and molecular assays. Combination studies were conducted using CHK1, ATR, and PARP-1 inhibitors.

**Results:**

Compounds **7b**, **7d**, and **7e** demonstrated the most potent antiproliferative activity, with GI_50_ values below 2.5 μM in leukemic cell lines. Compound **7e** exhibited notable cytotoxicity, with GI_50_ values of 1.1 μM (MV4-11) and 2.7 μM (K562). Compounds **7b** and **7e** significantly inhibited topoisomerase I activity and effectively suppressed topoisomerase IIα-mediated DNA relaxation. Cellular studies revealed S-phase cell cycle arrest, activation of apoptotic pathways (caspase cleavage and PARP-1 degradation), and induction of DNA damage response markers (γH2AX, p-CHK1, p53). In MV4-11 cells, combination treatment with CHK1 or ATR inhibitors resulted in pronounced synergistic cytotoxicity, whereas co-treatment with a PARP-1 inhibitor produced minimal synergy.

**Discussion:**

These findings identify bis-indole and pyrazolo[3,4-*b*]pyridine derivatives, particularly compound **7e**, as potent dual topoisomerase inhibitors with significant antileukemic activity. Their ability to induce DNA damage and enhance cytotoxicity in combination with DNA damage response inhibitors highlights their potential therapeutic value, especially in combination strategies targeting replication stress pathways in leukemia.

## Graphical Abstract

**Figure:**
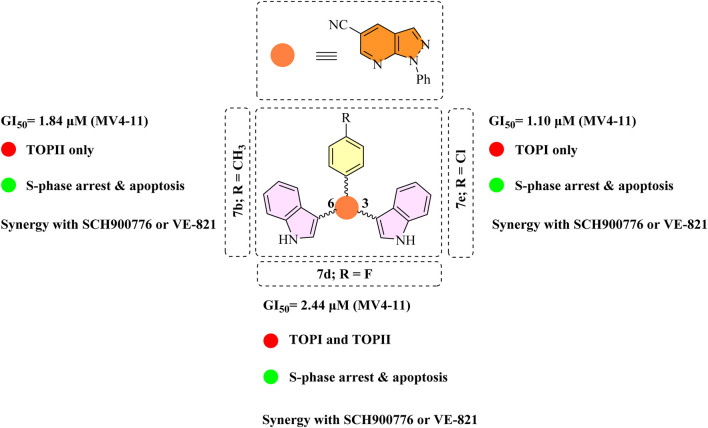

## Highlights

- Synthesized bis-indole and pyrazolo [3,4-*b*]pyridine derivatives exhibit potent anti-leukemic activity.
- Compounds 7b, 7d, and 7e strongly inhibit topoisomerase I and II-mediated DNA relaxation.
- Treatment induces S-phase arrest, apoptosis, and activation of DNA damage response in cells.
- Combinations with CHK1 and ATR inhibitors produce a synergistic cytotoxic effect in leukemic cells.

## Introduction

Leukemia is a broad category of haematological malignancies that are distinguished by the clonal proliferation of aberrant white blood cells within the peripheral blood and bone marrow (Tembhare et al., 2025). Current advances in genomic profiling have elucidated few of the key molecular drivers underlying leukemia subtypes, including BCR-ABL1 fusion in chronic myeloid leukemia (CML), FLT3 and NPM1 mutations in acute myeloid leukemia (AML), as well as dysregulated DNA damage response (DDR) pathways (Young et al., 2021; Popp et al., 2020). Among the critical regulators of DDR machinery, checkpoint kinase 1 (CHK1) and ataxia telangiectasia and Rad3-related protein (ATR) have emerged as pivotal targets (Wang et al., 2022). Notably, in the context of p53-deficient or chemotherapy-resistant leukemic cells, pharmacological inhibition of CHK1 or ATR has shown promise to potentiate cellular sensitivity to genotoxic stress, thereby offering a compelling therapeutic strategy for improving treatment outcomes (Komarova and Gudkov, 2001; Ghelli Luserna di Rorà et al., 2023).

DNA topoisomerases I and II (TOPI, TOPII) have long been recognized as key therapeutic targets due to the fundamental role of genomic instability in leukemogenesis (Baranello et al., 2025). By creating temporary single- or double-strand breaks in DNA, these enzymes mediate essential topological changes that facilitate transcription and replication (Otarbayev and Myung, 2024). In leukemia, their dysregulation contributes to chromosomal abnormalities and mutagenesis (Stoltze et al., 2025), and clinically, topoisomerase inhibitors, such as camptothecin (TOPI) and etoposide (TOPII), remain essential parts of leukemia treatment regimens (Lawal et al., 2021). Nonetheless, resistance development and dose-limiting toxicities underscore the need for novel strategies such as dual inhibition, targeting non-catalytic binding sites or combinatorial regimens with CHK1 or ATR inhibitors (Pfister and Ashworth, 2017).

**FIGURE 1:**
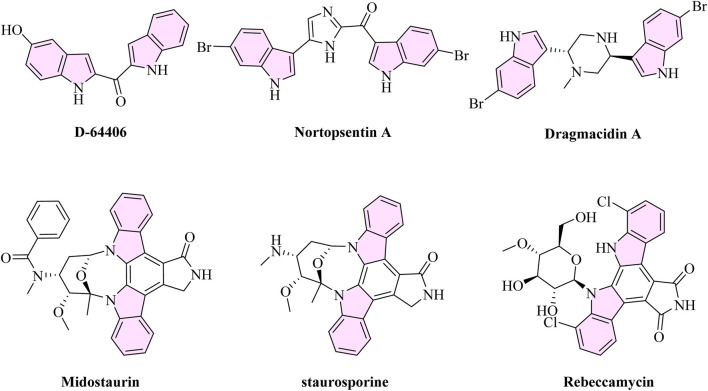
Structures of bis-indole derivatives alongside their anti-leukemic activity.

The structural diversity and polypharmacological potential of bis-indole-based compounds make them a promising class of anti-leukemic agents (Andreani et al., 2008). This scaffold can be found in a variety of forms, such as synthetic analogues (e.g., D-64406) (Teller et al., 2002; Sellmer et al., 2020), marine alkaloids (e.g., nortopsentin A), and natural compounds (e.g., dragmacidin A) (Xu et al., 2022). Interestingly, D-64406 has strong anti-AML effects as an FLT3 inhibitor. The FLT3-mutated AML is the target of the clinically approved multi-kinase inhibitor midostaurin, which has a bis-indole core (Wei and Malhotra, 2010). Furthermore, the indolocarbazole family members staurosporine and rebeccamycin (Long and Balasubramanian, 2000; Anizon et al., 1997) show kinase modulation and inhibition of topoisomerase II (Figure 1). These substances demonstrate the bis-indole motif’s therapeutic adaptability and underscore its ongoing significance in leukemia treatment development.

**FIGURE 2:**
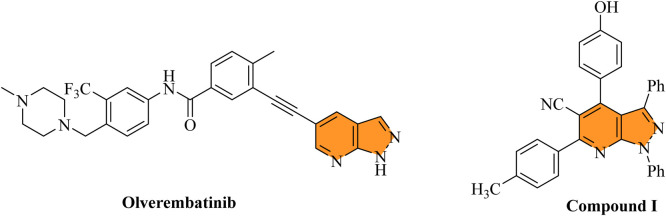
Structures of pyrazolo [3,4-*b*]pyridine derivatives alongside their anti-leukemic activity.

A pyrazole ring condensed with a pyridine ring forms the bicyclic fused heterocycle known as the pyrazolo [3,4-*b*]pyridine scaffold. The existence of this favored structure in a number of derivatives with strong anti-leukemic activity has drawn interest in leukemia research. Notably, substances like olverembatinib, a BCR-ABL inhibitor based on pyrazolo [3,4-*b*]pyridine, have shown notable effectiveness against drug-resistant mutations like T315I in CML cell lines (Jiang et al., 2022). Furthermore, a variety of pyrazolo [3,4-*b*]pyridine derivatives have demonstrated encouraging cytotoxicity against leukemia cell lines, including K562, MV4-11, and others. For example, compound I in recent investigations are examples of these derivatives (Barghash et al., 2022). These compounds frequently work by inhibiting kinases and interfering with signaling pathways that are essential for the survival and growth of leukemic cells (Figure 2). In the design of new anti-leukemia medicines, pyrazolo [3,4-*b*]pyridine is a useful structural motif since it can be used to create multifunctional inhibitors.

Despite significant advances in anti-leukemic therapy, many drugs are hindered by major limitations, such as dose-limiting toxicities, unfavorable side effects, drug resistance, and limited selectivity, leading to collateral damage to healthy tissues (Allegra et al., 2021). High-dose chemotherapy is frequently associated with the development of secondary cancers, myelosuppression, and cardiotoxicity (Monsuez et al., 2010). Additionally, leukemic cells often develop resistance through various mechanisms, including activation of alternative survival pathways, alterations in drug targets, and overexpression of drug efflux pumps (Zhang et al., 2019). Combination therapy approaches that use synergistic drug combinations at lower dosages have become more popular as a means of overcoming these obstacles (Sun et al., 2016).

In this study, we aimed to design and synthesize a set of thirteen new compounds that combine the pyrazolo [3,4-*b*]pyridine and bis-indole scaffolds. Particularly against DNA topoisomerases I and II, which are essential for leukemia cell survival and proliferation, these hybrid molecules have shown encouraging effectiveness. Our goal is to minimize frequent drawbacks such as drug resistance and dose-related toxicities while creating strong anti-leukemic medicines with improved efficacy through targeted topoisomerase inhibition. By concentrating on this mechanism, we hope to offer safer and more efficient therapeutic alternatives for the treatment of leukemia.

## Results and discussion

### Chemistry

**SCHEME 1:**
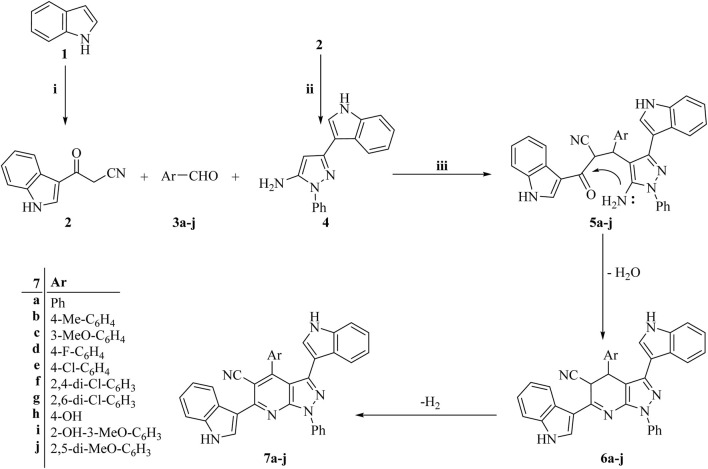
The synthetic route of target compounds (7a-j). Reagents and conditions: i. Cyanoacetic acid, acetic anhydride, 85 °C 30 min; ii. *Abs.* EtOH, C_6_H_5_NHNH_2_, reflux 1 h; iii. *Abs.* EtOH, reflux 12 h.

**SCHEME 2:**
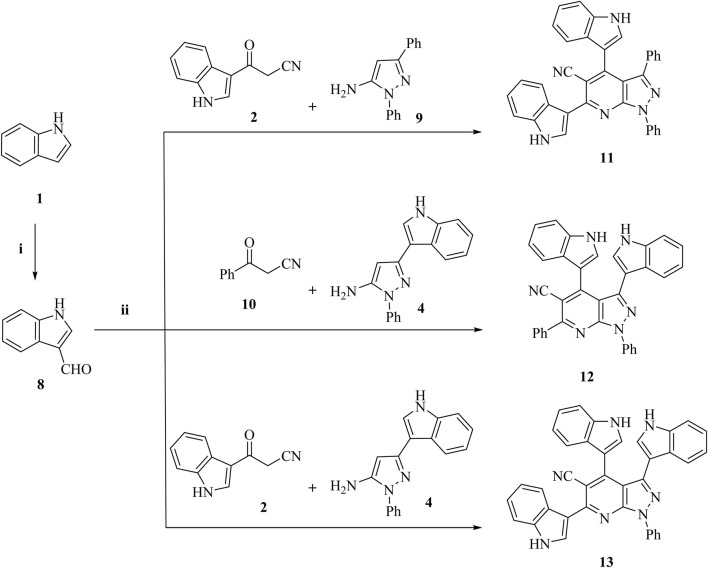
The synthetic route of target compounds (11–13). Reagents and conditions: i. POCl_3_, dry DMF, acid, 5 °C 30 min, then 35 °C 60 min; ii. *Abs.* EtOH, reflux 12 h.

Schemes 1, 2 show the synthetic routes taken to produce the target bis/tri-indolyl-conjugated pyrazolo [3,4-*b*]pyridine derivatives (7a-j, and 11–13). In order to get 3-(1*H*-indol-3-yl)-3-oxopropanenitrile (2), indole (1) was heated with cyanoacetic acid, while acetic anhydride was involved (Eldehna et al., 2024a).

Additionally, 3-(1*H*-indol-3-yl)-3-oxopropanenitrile (2) was condensed with phenylhydrazine in refluxing absolute ethanol to create the 3-substituted-1-phenyl-1*H*-pyrazol-5-amine (4) (Barghash et al., 2022; Eldehna et al., 2024b).

As shown in Schemes 1, 2, the target bis- and tris-indoles that hybrid to pyrazolo [3,4-*b*]pyridine derivatives (7a-j, and 11–13) were then made using a one-pot, three-component reaction that included the relevant 3-substituted-1-phenyl-1*H*-pyrazol-5-amine (4 or 9), an equimolar amount of the corresponding 3-oxo-3-arylpropanenitrile (2 or 10), and the appropriate aldehyde (3a-j and 8).

Intermediates 5a-j are produced as part of the reaction mechanism, and they are cyclized and then water-eliminated to produce intermediates 6a-j. The required compounds 7a-j are subsequently obtained through a final dehydrogenation process. High-resolution mass spectrometry (HRMS) and nuclear magnetic resonance (NMR) spectroscopy (^1^H and ^13^C) validated the synthesized compounds’ structures, which were in agreement with the suggested structures.

### Structure elucidation of the target compounds

The ^1^H NMR spectrum data of target compounds 7a-j and 11–13 verified the validity of their structures. In particular, the absence of the aldehydic proton (CH = O) signals from aldehydes (3a-j and 8) and the active methylene protons (CH_2_) from nitriles (2 and 10) around δ 3.5 ppm, as well as the disappearance of characteristic signals around δ 5.0 ppm, which correspond to the two NH_2_ protons of the precursor 3-substituted-1-phenyl-1*H*-pyrazol-5-amines (4 and 9). The addition of aromatic moieties throughout the process was further supported by the spectra, which showed an increase in aromatic proton signals.

The suggested structures were further supported by the ^13^C NMR spectra, which revealed the elimination of carbon signals linked to nitrile and aldehyde carbonyl groups, which are normally detected at δ 190 ppm. These modifications verified that the initial materials had completely changed into the final pyrazolo [3,4-*b*]pyridine derivatives.

With variances falling within the permissible range of ±0.4%, the findings of the elemental analysis showed good agreement with the estimated values for the target compounds’ molecular formulae. The structures were further validated by molecular ion peaks obtained from high-resolution mass spectrometry (HRMS) that matched the predicted molecular weights. The effectiveness and selectivity of the synthetic processes were further demonstrated by high-performance liquid chromatography (HPLC) analysis, which verified that all synthesized compounds had a purity higher than 95.00%.

### Biological evaluation

#### Anti-proliferative activity

First, four cancer cell lines, acute myeloid leukemia (MV4-11), chronic myeloid leukemia (K562), melanoma (G361), and non-small cell lung cancer (HCC827), were used to assess the anti-proliferative activity of recently synthesized derivatives (Table 1). Several tested compounds demonstrated significant cytotoxicity, especially leukemic cells. In contrast, the G361 cell line exhibited low overall sensitivity, with most compounds showing GI_50_ values above 8 µM or no detectable action at the tested dosages. Similarly, the non-small cell lung cancer cell line HCC827 showed no significant cytotoxic response, with GI_50_ exceeding 10 µM (data not shown).

**TABLE 1:** *In vitro* anti-proliferative activity of target compounds 7a-j and 11–13 against MV4-11 and K562 (leukemia) and G361 (melanoma) cell lines.

Compound	GI_50_ (µM)^a^ ± SD		
	MV4-11	K562	G361
	(Leukemia cell line)	(Leukemia cell line)	(Melanoma cell line)
**7a**	>10	>10	>10
**7b**	1.84 ± 0.37	2.77 ± 0.08	8.71 ± 1.01
**7c**	5.64 ± 1.60	2.43 ± 0.21	>10
**7d**	2.44 ± 0.18	2.43 ± 0.28	9.76 ± 0.05
**7e**	1.10 ± 0.01	2.70 ± 0.30	>10
**7f**	3.69 ± 0.13	6.54 ± 0.68	>10
**7g**	8.07 ± 1.37	8.32 ± 0.02	>10
**7h**	6.98 ± 2.49	2.53 ± 0.03	7.93 ± 0.36
**7i**	3.40 ± 0.14	5.21 ± 1.43	8.08 ± 0.24
**7j**	8.73 ± 0.37	>10	9.15 ± 0.90
**11**	>10	>10	>10
**12**	5.57 ± 1.63	>10	8.69 ± 0.41
**13**	>10	>10	>10

^a^ Assayed in triplicate.

Among the tested derivatives, 7b, 7d, and 7e exhibited the most potent anti-proliferative activity with GI_50_ values below 2.50 μM in MV4-11 cells. In particular, compound 7b, with GI_50_ values of 1.10 µM for MV4-11 and 2.70 µM for K562, emerged as the most active compound in this series. In contrast, G361 showed no significant response (GI_50_ > 10 µM). Likewise, 7b and 7d showed limited efficacy against G361, but they maintained substantial activity in MV4-11 (1.84 µM and 2.44 µM, respectively) and K562 (2.77 µM and 2.43 µM, respectively).

**FIGURE 3:**
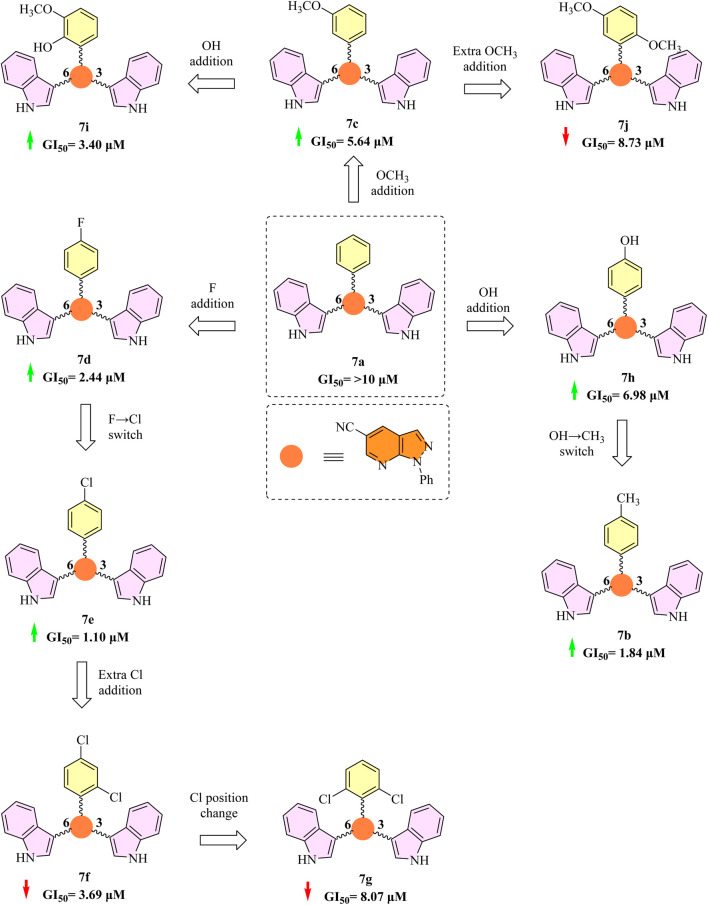
The 3,6-bi-indole target compounds’ structure-activity relationship (SAR) as *in vitro* anti-cancer agents based on their inhibition on the MV4-11 cell line.

In comparison to the unsubstituted phenyl analogue 7a (GI_50_ > 10 μM; Figure 3), the addition of one or more halogen atoms onto the phenyl ring had varying impacts on the inhibitory activity of 3,6-bi-indole derivatives (7a-j) when tested against MV4-11 cell lines.

Compound 7d (GI_50_ = 2.44 µM) showed a significant increase in activity with the substitution of a fluorine atom at the para-position. As seen in 7e (GI_50_ = 1.10 µM), the activity was further increased by about twofold by substituting a chlorine atom for the fluorine at the same location. These discrepancies might be explained by variations in the atomic radius of the halogens as well as their electrical and lipophilic characteristics, which could affect the binding affinity to the target site.

However, adding a second chlorine atom at the *ortho*-position (compound 7f) significantly decreased activity (GI_50_ = 3.69 µM), suggesting that there may be unfavorable electrical interactions or steric hindrance. With GI_50_ = 8.07 µM, a further decrease in activity was noted when the *para*-chlorine was moved to a second *ortho*-position (compound 7g), indicating that *ortho*-substitution with large halogens may obstruct optimal binding.

Activity was also affected by the addition of methoxy groups that donate electrons. Activity was moderately improved when a single *meta*-methoxy group was added to parent drug 7a (compound 7c, GI_50_ = 5.64 µM). However, adding a second methoxy group (compound 7j) reduced activity (GI_50_ = 8.73 µM), most likely as a result of steric effects or excessive electron donation. However, compound 7i, which added a hydroxy group in place of a second methoxy, showed a significant increase in activity (GI_50_ = 3.40 µM), underscoring the significance of hydrogen-bonding capacity.

It's interesting to note that compound 7h’s increased activity (GI_50_ = 6.98 µM) was achieved by substituting this strongly donating group with a hydroxyl group (OH), which has a lesser electron-donating nature. By substituting the methyl group (CH_3_), an even less electron-donating substituent, for the OH group in compound 7b, this tendency was further confirmed. This led to a considerable increase in activity (GI_50_ = 1.84 µM), which was a four-fold improvement over 7h (Figure 3).

As demonstrated by the parent molecule 7a, which likewise has two phenyl rings, all of the previously listed SAR compounds have a structural scaffold that contains indole moieties at positions 3 and 6. No discernible increase in inhibitory action was shown when one of the indole units was substituted for the phenyl ring, putting the indole groups at positions 4 and 6. This was demonstrated in compound 11 (GI_50_ > 10 µM). As seen in compound 12 (GI_50_ = 5.57 µM), the activity significantly increased when the indole rings were placed at positions 3 and 4, indicating a favorable spatial orientation or electronic effect in this arrangement. It's interesting to note that compound 13 (GI_50_ > 10 µM) did not exhibit any additional boost in activity when a third indole ring was added in place of one of the phenyl rings, putting indole moieties at positions 3, 4 and 6. This result suggests that the precise placement of these rings is more important for improving activity than merely increasing the number of indole units.

#### Topoisomerase relaxation assay

**FIGURE 4:**
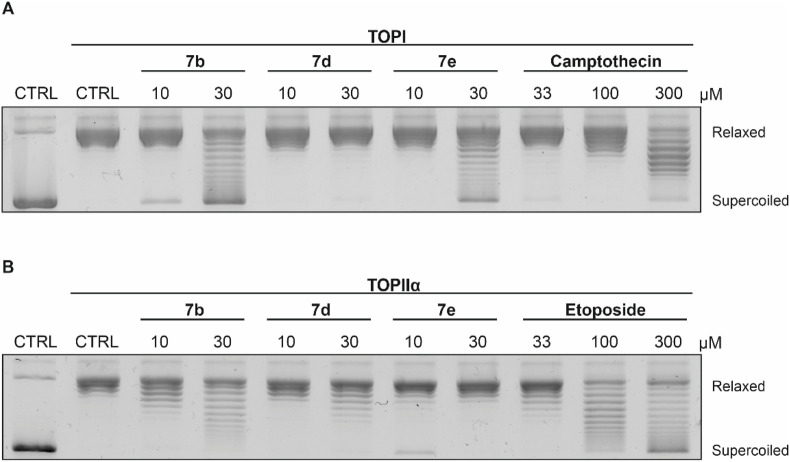
Inhibitory effects of 7b, 7d, and 7e on the DNA relaxation activity of **(A)** TOPI and **(B)** TOPIIα. Camptothecin and etoposide were included as positive controls.

To evaluate whether the target molecules act as topoisomerase inhibitors, DNA relaxation assays were conducted with the most potent derivatives 7b, 7d, and 7e. Camptothecin and etoposide were included as positive controls, representing established inhibitors of TOPI and TOPII, respectively (Yakkala et al., 2023). As shown in Figure 4A, 7b and 7e demonstrated dose-dependent inhibition of TOPI-mediated DNA relaxation, while 7d showed no relaxation of supercoiled DNA at both tested concentrations (10 and 30 μM). In the TOPIIα relaxation assay (Figure 4B), 7b and 7d exhibited very potent inhibition of DNA relaxation, while 7e showed only reduced efficacy in lower tested concentrations. Positive controls, camptothecin and etoposide, confirmed the validity of the assay conditions. Collectively, these results indicate that the tested compounds inhibit the relaxation ability of TOPI and/or TOPIIα with varying degrees of potency.

#### Immunodetection and cell cycle analysis of MV4-11

The most potent compounds, 7b, 7d and 7e, were further analyzed for their cellular effects in MV4-11 cells treated for 24 h with the indicated concentrations. The treatment resulted in rapid phosphorylation of key markers of the DNA damage response, including histone H2AX (S139), CHK1 (S317), and p53 (S15) (Zhao et al., 2008), indicating the induction of DNA damage consistent with topoisomerase inhibition. These lesions also led to a concentration-dependent accumulation of cyclin E, consistent with delayed S-phase progression and impaired cell cycle control.

**FIGURE 5:**
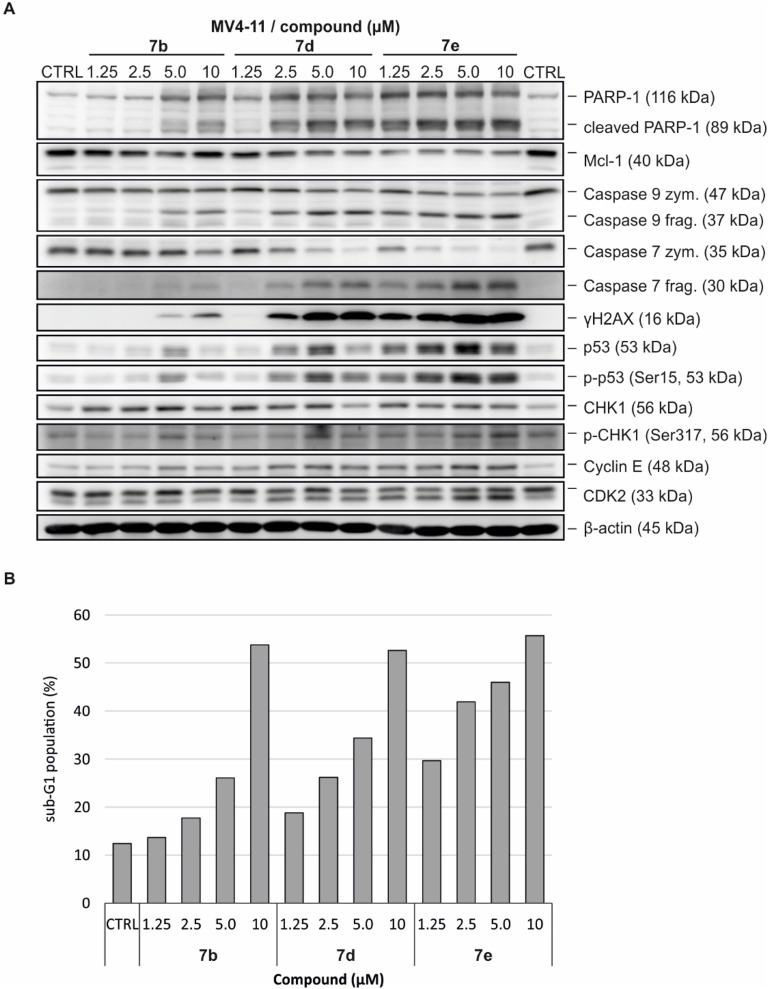
Cellular effects of compounds 7b, 7d, and 7e on MV4-11 cells treated for 24 h. **(A)** Immunoblotting of markers of cell death and DNA damage. β-actin was detected as a loading control. **(B)** Analysis of induction of cell death (sub-G1 population).

As the cellular response progressed, immunoblot analysis revealed activation of proapoptotic pathways, with strong cleavage of initiator caspase-9 and executioner caspase-7 (Fiandalo and Kyprianou, 2012). This was accompanied by robust cleavage of PARP-1 and downregulation of the anti-apoptotic protein Mcl-1, particularly in response to compound 7e (Figure 5A; Supplementary Figure S33). To confirm the induction of cell death, a cell cycle analysis of MV4-11 cells treated with the tested compounds was performed. Consistent with previous findings, all three derivatives induced a dose-dependent accumulation of cells in the S phase, indicative of replication stress or S-phase arrest, followed by a pronounced increase in the sub-G1 population, reflecting apoptotic cell death (Figure 5B), comparable to the effects observed with camptothecin and etoposide (Supplementary Figure S34). Collectively, the effects of compounds 7b, 7d, and 7e on markers of DNA damage and apoptosis were comparable to those induced by known topoisomerase inhibitors.

#### Synergistic effect of combined treatment

Dose-limiting toxicities and the development of drug resistance frequently restrict the therapeutic efficacy of topoisomerase inhibitors in the treatment of malignancies, particularly acute myeloid leukemia (AML). Combinatorial approaches have been studied to get around these restrictions, especially those that target complementary oncogenic pathways and take advantage of synthetic lethality. Given the close link between topoisomerase inhibition and DDR activation, combining topoisomerase inhibitors with DDR-targeting agents represents a rational approach to enhance both therapeutic efficacy and durability. To further explore this therapeutic rationale, we investigated whether combining the most active topoisomerase inhibitors with selected DDR-targeting agents could enhance anti-leukemic efficacy.

**FIGURE 6:**
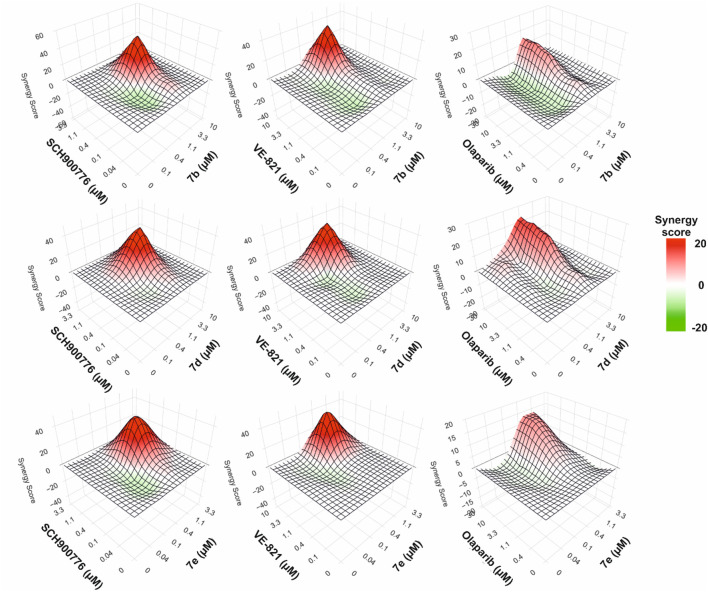
Drug synergy assessment of combined treatment with 7b, 7d, and 7e in combination with SCH900776, VE-821 or olaparib after 72-h incubation in MV4-11 cells. Heatmaps were generated using the Highest single agent (HSA) model in Synergy Finder (3.0) to calculate synergy scores based on representative duplicate data from one of two independent experiments. Synergistic and antagonistic dose ratio regions are marked in red and green, respectively.

When combined with specific DDR inhibitors, such as SCH900776 (a CHK1 inhibitor) (Guzi et al., 2011), VE-821 (an ATR inhibitor) (Reaper et al., 2011), and olaparib (a PARP-1 inhibitor) (Fo et al., 2009), we assessed the synergistic potential of the most potent topoisomerase-targeting derivatives (7b, 7d, and 7e). After 72 h of incubation, these combinations were examined in MV4-11 AML cells to ascertain how they affected interaction dynamics and cell viability (Figure 6). Pharmacological drug interaction analysis was performed using SynergyFinder 3.0, which calculated synergy scores across a matrix of drug concentrations based on the Highest Single Agent (HSA) model. The interaction landscapes were visualized as heatmaps, where green areas indicated antagonism and red areas indicated synergistic interactions, where the combined effect is greater than the highest single agent.

When paired with either SCH900776 or VE-821, all three derivatives showed strong synergy, indicating a functional interaction between checkpoint kinase blockade and topoisomerase inhibition. These findings provide credence to the theory that cytotoxicity in AML cells can be markedly increased by targeting several nodes in the DNA damage response system, specifically checkpoint abrogation and DNA strand break accumulation.

Olaparib combination therapies, on the other hand, produced very little improvement over the effects of the individual drugs and just a slight synergy. This result implies that PARP inhibition might not be as useful as CHK1 or ATR inhibition in enhancing the mechanism of action of these compounds. The drugs’ specificity in interacting with the ATR–CHK1 axis is highlighted by the differential synergy profiles, which also support the idea that certain combinations should be given priority in upcoming preclinical research (Figure 6).

### Docking

**FIGURE 7:**
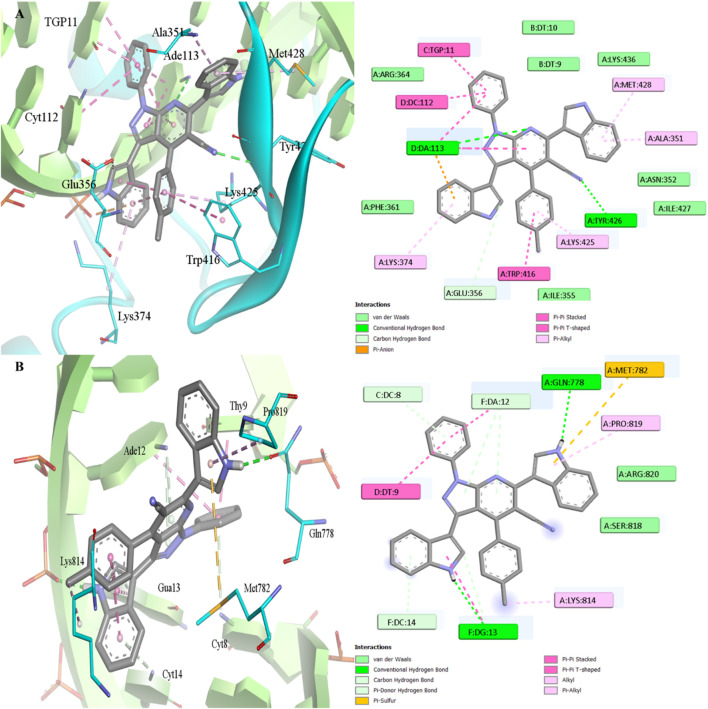
Molecular docking of 7b inside the active site of **(A)** TOPI (PDB: 1K4T) with docking score −10.5 kcal/mol and **(B)** TOPII (PDB: 3QX3) with docking score −11.0 kcal/mol.

The structure of 7b was subjected to molecular docking modelling in order to assess the potential mode of binding inside TOPI and TOPII. The docking protocol was found to be valid as indicated from the low RMSD of docked poses from the coordinate of crystallized ligands of TOPI (0.760 Å) and TOPII (0.350 Å) (Mishra et al., 2021; Mishra and Sharma, 2016), as seen in Supplementary Figure S35. The intercalation was observed for the cocrystal with the DNA bases DT10, TGP11, DC112, and DC113 inside TOPI, while bases DC8, DA12, and DG13 were involved inside TOPII. Hydrogen bonding contributed to the ligand’s affinity through three H-bonds for TOPI and four H-bonds for TOPII. The aromatic system of both ligands was involved in hydrophobic interaction beside π–π interactions. As anticipated from the best docking pose, 7b fits well into the major groove region of the DNA double helix (Figure 7). The intercalation was not complete due to the steric structure of 7b while the phenyl and one of the indole rings were observed to engage by π–π interactions with the same bases observed with crystallized ligands. This was reflected by the good docking scores achieved by 7b and 7e inside TOPI (−10.5 kcal/mol and −11.2 kcal/mol) and TOPII (−11.0 kcal/mol and −11.0 kcal/mol) which were lower than the reference ligands inside TOPI and TOPII with docking scores −12.6 kcal/mol and −14.7 kcal/mol, respectively. In addition, H-bonding was also observed for 7b with two H- bonds inside both TOPI (DA 113 and Tyr426) and TOPII (DG13 and Gln778). Furthermore, one of the indole rings made π-anion interaction with DA113 inside TOPI while π-sulfur interaction was observed with Met782 inside TOPII. Interestingly, inside the TOPI tolyl ring was involved in π-π interaction with Tyr416 and hydrophobic interaction with Lys425, Ile377 while it makes only hydrophobic interaction with Lys814 inside TOPII. Similarly, compound 7e exhibited the same interactions as 7b, except that in TOPI the chlorophenyl ring formed hydrophobic contacts with Ile377, while DA113 did not participate in hydrogen bonding with the pyridine ring (Supplementary Figure S36). These interactions may attribute the favoured activity of 7b and 7e inside Both TOPI and TOPII.

## Conclusion

The novel series of bis(indolyl)-conjugated pyrazolo [3,4-*b*]pyridine derivatives exhibited potent anti-proliferative activity against the leukemic cell lines MV4-11 and K562. Among the tested compounds, derivatives 7b, 7d, and 7e emerged as the most effective, with GI_50_ values in the low micromolar range. Topoisomerase relaxation assays confirmed that these derivatives inhibit the relaxation of supercoiled DNA mediated by topoisomerase I and/or topoisomerase IIα, with varying levels of potency. Furthermore, compounds 7b, 7d, and 7e induced DNA damage characteristics for cellular responses to topoisomerase inhibition. This was also confirmed by cellular studies comparing their molecular effects to those of known topoisomerase inhibitors, camptothecin, and etoposide. The DNA damage induced by these derivatives led to cell cycle disruption and subsequent cell death, as evidenced by strong cleavage of PARP-1 and caspases. Additionally, combinatorial treatment experiments demonstrated a synergistic potential of compounds 7b, 7d, and 7e when combined with inhibitors of DNA damage response pathways, such as CHK1 and ATR inhibitors, resulting in enhanced cytotoxic effects.

## Experimental

### Chemistry

#### General

Commercial suppliers provided all of the materials, which were used exactly as supplied. Silica gel plates were subjected to TLC in order to track the purity and development of the reaction. Uncorrected melting points were measured, and standard equipment was used for elemental analysis, FTIR, and NMR (^1^H, ^13^C, and DEPT-135). A Bruker MicroTOF spectrometer was used to record high-resolution mass spectra. As stated in the literature, starting ingredients 2 (Eldehna et al., 2024a), 4 (Eldehna et al., 2024a), and 8 (Liu et al., 2019) were synthesized. A ZORBAX Eclipse Plus C18 column was used for HPLC analysis, which verified that the produced chemicals were more than 95% pure.

#### General procedure for synthesis of target compounds (7a-j, and 11–13)

TLC was used to track the reaction progress of a combination of different aldehydes (3a-j and 8), equimolar quantities of 3-oxo-3-arylpropanenitriles (2 and 10), and 3-substituted-1-phenyl-1*H*-pyrazol-5-amines (4 and 9) that were refluxed in 20 mL of absolute ethanol for 12 h. The reaction mixture was finished and then left to cool to room temperature. Filtration, air drying, and recrystallization of the resultant precipitate from ethanol yielded the required, highly pure target compounds (7a-j, and 11–13).

#### 3,6-Di (1H-Indol-3-yl)-1,4-diphenyl-1H-pyrazolo [3,4-b]pyridine-5-carbonitrile (7a)

Yield (77%) as a yellow powder, with Mp: >300 °C. ^1^H NMR (700 MHz, DMSO*d*_6_) δ (ppm): 12.18 (s, 2H, 2NH), 8.38 (s, 4H, Ar-Hs), 8.15 (dt, *J* = 7.5, 1.0 Hz, 4H, Ar-Hs), 7.52 (dt, *J* = 8.1, 1.0 Hz, 4H, Ar-Hs), 7.27 – 7.22 (m, 8H, Ar-Hs). ^13^C NMR (176 MHz, DMSO*d*_6_) δ (ppm): 172.43, 137.05, 135.90, 125.58, 123.73, 122.75, 121.43, 116.85, 114.86, 112.83, 99.90, 83.64. HRMS (ESI): *m*/*z*: [M + H]^+^ calcd. 527.1979 and found 527.1975. Anal. Calcd. (Found) For C_35_H_22_N_6_: C, 79.83 (80.02); H, 4.21 (4.20); N, 15.96 (16.05)%.

#### 3,6-Di (1H-Indol-3-yl)-1-phenyl-4-(p-tolyl)-1H-pyrazolo [3,4-b]pyridine-5-carbonitrile (7b)

Yield (80%) as a yellow powder, with Mp: >300 °C. HPLC: R_T_ 5.165 min (purity: 99.18%). ^1^H NMR (700 MHz, DMSO*d*_6_) δ (ppm): 11.67 (d, *J* = 2.8 Hz, 1H, NH), 11.19 (d, *J* = 2.6 Hz, 1H, NH), 8.32 (dd, *J* = 7.9, 1.2 Hz, 1H, Ar-H), 7.87 – 7.85 (m, 2H, Ar-Hs), 7.80 (d, *J* = 2.8 Hz, 1H, Ar-H), 7.63 – 7.59 (m, 3H, Ar-Hs), 7.49 (dt, *J* = 8.2, 1.0 Hz, 1H, Ar-H), 7.41 (tt, *J* = 7.4, 1.2 Hz, 1H, Ar-H), 7.37 (d, *J* = 2.7 Hz, 1H, Ar-H), 7.34 – 7.32 (m, 3H, Ar-Hs), 7.19 (ddd, *J* = 8.2, 7.0, 1.2 Hz, 1H, Ar-H), 7.18 – 7.11 (m, 4H, Ar-Hs), 7.06 (ddd, *J* = 8.0, 6.9, 1.1 Hz, 1H, Ar-H), 2.26 (s, 3H, CH_3_). ^13^C NMR (176 MHz, DMSO*d*_6_) δ (ppm): 172.48, 145.19, 144.34, 143.13, 139.30, 138.32, 136.42, 136.24, 136.22, 129.75, 129.58, 128.39, 128.03, 126.95, 125.86, 125.67, 125.12, 123.23, 122.43, 122.30, 122.13, 120.39, 120.36, 120.27, 119.90, 112.52, 111.71, 109.01, 108.13, 100.01, 83.86, 21.08.HRMS (ESI): *m*/*z*: [M + H]^+^ calcd. 541.2135 and found 541.2133. Anal. Calcd. (Found) For C_36_H_24_N_6_: C, 79.98 (80.17); H, 4.47 (4.45); N, 15.55 (15.46)%.

#### 3,6-Di (1H-Indol-3-yl)-4-(3-methoxyphenyl)-1-phenyl-1H-pyrazolo [3,4-b]pyridine-5-carbonitrile (7c)

Yield (85%) as a yellow powder, with Mp: >300 °C. ^1^H NMR (500 MHz, DMSO*d*_6_) δ (ppm): 11.68 (d, *J* = 2.8 Hz, 1H, NH), 11.24 (d, *J* = 2.6 Hz, 1H, NH), 8.36 (d, *J* = 2.2 Hz, 2H, Ar-Hs), 8.31 – 8.29 (m, 1H, Ar-H), 7.85 – 7.80 (m, 3H, Ar-Hs), 7.62 – 7.60 (m, 1H, Ar-H), 7.58 – 7.54 (m, 2H, Ar-Hs), 7.50 – 7.43 (m, 4H, Ar-Hs), 7.38 – 7.31 (m, 2H, Ar-Hs), 7.18 – 7.14 (m, 1H, Ar-H), 7.01 – 6.97 (m, 2H, Ar-Hs), 6.75 (ddd, *J* = 8.3, 2.6, 1.0 Hz, 1H, Ar-H), 3.67 (s, 3H, OCH_3_). HRMS (ESI): *m*/*z*: [M + H]^+^ calcd. 557.2084 and found 557.2083. Anal. Calcd. (Found) For C_36_H_24_N_6_O: C, 77.68 (77.86); H, 4.35 (4.33); N, 15.10 (15.04)%.

#### 4-(4-Fluorophenyl)-3,6-di (1H-indol-3-yl)-1-phenyl-1H-pyrazolo [3,4-b]pyridine-5-carbonitrile (7d)

Yield (86%) as a yellow powder, with Mp: >300 °C. HPLC: R_T_ 8.258 min (purity: 99.67%). ^1^H NMR (700 MHz, DMSO*d*_6_) δ (ppm): 11.68 (d, *J* = 2.8 Hz, 1H, NH), 11.21 (d, *J* = 2.7 Hz, 1H, NH), 8.30 (d, *J* = 7.9 Hz, 1H, Ar-H), 7.87 – 7.85 (m, 2H, Ar-Hs), 7.82 (d, J = 2.8 Hz, 1H, Ar-H), 7.62 – 7.59 (m, 3H, Ar-Hs), 7.48 (dt, *J* = 8.6, 6.5 Hz, 3H, Ar-Hs), 7.42 – 7.40 (m, 1H, Ar-H), 7.39 (d, *J* = 2.7 Hz, 1H, Ar-H), 7.34 (d, *J* = 8.0 Hz, 1H, Ar-H), 7.19 – 7.11 (m, 5H, Ar-Hs), 7.06 (ddd, *J* = 7.9, 6.8, 1.1 Hz, 1H, Ar-H). ^13^C NMR (176 MHz, DMSO*d*_6_) δ (ppm): 172.44, 162.11, 160.73, 145.15, 144.54, 142.24, 142.23, 139.25, 138.31, 136.43, 136.23, 129.95, 129.90, 129.74, 128.48, 127.01, 125.84, 125.64, 125.11, 123.32, 122.31, 122.29, 122.14, 120.39, 120.25, 119.90, 115.77, 115.65, 112.53, 111.72, 108.89, 108.02, 99.68, 83.43. HRMS (ESI): *m*/*z*: [M + H]^+^ calcd. 545.1884 and found 545.1875. Anal. Calcd. (Found) For C_35_H_21_FN_6_: C, 77.19 (77.03); H, 3.89 (3.91); N, 15.43 (15.39)%.

#### 4-(4-Chlorophenyl)-3,6-di (1H-indol-3-yl)-1-phenyl-1H-pyrazolo [3,4-b]pyridine-5-carbonitrile (7e)

Yield (90%) as a yellow powder, with Mp: >300 °C. HPLC: R_T_ 10.632 min (purity: 99.55%). ^1^H NMR (700 MHz, DMSO*d*_6_) δ (ppm): 11.70 (d, *J* = 2.9 Hz, 1H, NH), 11.22 (d, *J* = 2.6 Hz, 1H, NH), 8.31 – 8.30 (m, 1H, Ar-H), 7.87 – 7.85 (m, 2H, Ar-Hs), 7.83 (d, *J* = 2.8 Hz, 1H, Ar-H), 7.63 – 7.59 (m, 3H, Ar-Hs), 7.49 (dt, *J* = 8.1, 1.0 Hz, 1H, Ar-H), 7.48 – 7.45 (m, 2H, Ar-Hs), 7.43 – 7.40 (m, 3H, Ar-Hs), 7.39 (d, *J* = 2.7 Hz, 1H, Ar-H), 7.35 (dt, *J* = 8.1, 1.0 Hz, 1H, Ar-H), 7.20 (ddd, *J* = 8.2, 7.0, 1.3 Hz, 1H, Ar-H), 7.16 (ddd, *J* = 8.0, 7.0, 1.2 Hz, 1H, Ar-H), 7.12 (ddd, *J* = 8.1, 6.9, 1.3 Hz, 1H, Ar-H), 7.07 (ddd, *J* = 8.0, 6.9, 1.1 Hz, 1H, Ar-H). ^13^C NMR (176 MHz, DMSO*d*_6_) δ (ppm): 172.53, 145.22, 144.94, 144.79, 139.27, 138.42, 136.49, 136.29, 131.85, 131.80, 130.01, 129.81, 129.04, 128.60, 127.12, 125.88, 125.68, 125.15, 123.41, 122.40, 122.34, 122.32, 122.24, 120.49, 120.30, 119.99, 112.60, 111.81, 108.89, 108.04, 99.41, 83.10. HRMS (ESI): *m*/*z*: [M + H]^+^ calcd. 561.1589 and found 561.1589. Anal. Calcd. (Found) For C_35_H_21_ClN_6_: C, 74.93 (75.12); H, 3.77 (3.80); N, 14.98 (15.06)%.

#### 4-(2,4-Dichlorophenyl)-3,6-di (1H-indol-3-yl)-1-phenyl-1H-pyrazolo [3,4-b]pyridine-5-carbonitrile (7f)

Yield (89%) as a yellow powder, with Mp: >300 °C. ^1^H NMR (700 MHz, DMSO*d*_6_) δ (ppm): 11.70 (d, *J* = 2.9 Hz, 1H, NH), 11.33 (d, *J* = 2.7 Hz, 1H, NH), 8.26 (dd, *J* = 8.0, 1.1 Hz, 1H, Ar-H), 7.86 – 7.84 (m, 3H, Ar-Hs), 7.65 – 7.64 (m, 2H, Ar-Hs), 7.62 – 7.59 (m, 2H, Ar-Hs), 7.49 – 7.47 (m, 2H, Ar-Hs), 7.43 – 7.40 (m, 2H, Ar-Hs), 7.35 (dt, *J* = 8.1, 1.0 Hz, 1H, Ar-H), 7.30 (d, *J* = 2.7 Hz, 1H, Ar-H), 7.20 (ddd, *J* = 8.1, 7.0, 1.3 Hz, 1H, Ar-H), 7.16 (ddd, *J* = 8.1, 7.0, 1.2 Hz, 1H, Ar-H), 7.13 (ddd, *J* = 8.1, 6.9, 1.3 Hz, 1H, Ar-H), 7.06 (ddd, *J* = 8.0, 7.0, 1.1 Hz, 1H, Ar-H). ^13^C NMR (176 MHz, DMSO*d*_6_) δ (ppm): 172.44, 145.61, 144.85, 141.77, 139.13, 138.88, 136.42, 136.30, 133.07, 132.66, 129.79, 129.74, 129.30, 128.63, 127.17, 125.85, 125.60, 124.08, 123.49, 122.34, 122.29, 122.21, 121.62, 120.45, 120.27, 119.97, 112.53, 111.78, 108.64, 108.12, 98.51, 81.15. Anal. Calcd. (Found) For C_35_H_20_Cl_2_N_6_: C, 70.60 (70.38); H, 3.39 (3.40); N, 14.11 (14.04)%.

#### 4-(2,6-Dichlorophenyl)-3,6-di (1H-indol-3-yl)-1-phenyl-1H-pyrazolo [3,4-b]pyridine-5-carbonitrile (7 g)

Yield (82%) as a yellow powder, with Mp: >300 °C. ^1^H NMR (700 MHz, DMSO*d*_6_) δ (ppm): 11.67 (d, *J* = 2.8 Hz, 1H, NH), 11.28 (d, *J* = 2.6 Hz, 1H, NH), 8.18 – 8.16 (m, 1H, Ar-H), 7.84 (d, *J* = 2.8 Hz, 1H, Ar-H), 7.79 – 7.77 (m, 2H, Ar-Hs), 7.67 – 7.66 (m, 1H, Ar-H), 7.60 – 7.57 (m, 2H, Ar-Hs), 7.53 (dd, *J* = 8.1, 1.3 Hz, 1H, Ar-H), 7.49 (dt, *J* = 8.1, 1.0 Hz, 1H, Ar-H), 7.41 – 7.39 (m, 2H, Ar-Hs), 7.33 (dt, *J* = 8.2, 0.9 Hz, 1H, Ar-H), 7.28 (t, *J* = 8.0 Hz, 1H, Ar-H), 7.20 (d, *J* = 2.4 Hz, 1H, Ar-H), 7.19 – 7.18 (m, 1H, Ar-H), 7.16 (ddd, *J* = 8.0, 7.0, 1.2 Hz, 1H, Ar-H), 7.11 (ddd, *J* = 8.1, 6.9, 1.2 Hz, 1H, Ar-H), 7.03 (ddd, *J* = 7.9, 6.9, 1.1 Hz, 1H, Ar-H). ^13^C NMR (176 MHz, DMSO*d*_6_) δ (ppm): 172.44, 147.38, 144.76, 139.72, 139.12, 137.11, 136.99, 136.96, 136.39, 136.31, 134.97, 131.62, 130.04, 129.73, 128.89, 128.23, 127.14, 125.92, 125.87, 123.86, 123.52, 122.28, 122.17, 122.05, 121.33, 120.39, 120.32, 119.82, 112.49, 111.68, 108.78, 108.19, 96.81, 78.52. HRMS (ESI): *m*/*z*: [M + H]^+^ calcd. 595.1199 and found 595.1199. Anal. Calcd. (Found) For C_35_H_20_Cl_2_N_6_: C, 70.60 (70.35); H, 3.39 (3.42); N, 14.11 (14.16)%.

#### 4-(4-Hydroxyphenyl)-3,6-di (1H-indol-3-yl)-1-phenyl-1H-pyrazolo [3,4-b]pyridine-5-carbonitrile (7 h)

Yield (79%) as a yellow powder, with Mp: >300 °C. ^1^H NMR (700 MHz, DMSO*d*_6_) δ (ppm): 11.66 (d, *J* = 2.8 Hz, 1H, NH), 11.19 (d, *J* = 2.7 Hz, 1H, NH), 8.33 – 8.31 (m, 1H, Ar-H), 7.86 – 7.85 (m, 2H, Ar-Hs), 7.80 (d, *J* = 2.8 Hz, 1H, Ar-H), 7.62 – 7.58 (m, 3H, Ar-Hs), 7.48 (dt, *J* = 8.2, 1.0 Hz, 1H, Ar-H), 7.40 (td, *J* = 7.4, 1.2 Hz, 1H, Ar-H), 7.34 (dt, *J* = 8.1, 1.0 Hz, 1H, Ar-H), 7.31 (d, *J* = 2.6 Hz, 1H, Ar-H), 7.24 – 7.22 (m, 2H, Ar-Hs), 7.19 (ddd, *J* = 8.2, 7.0, 1.2 Hz, 1H, Ar-H), 7.13 (dddd, *J* = 13.7, 8.1, 6.9, 1.2 Hz, 2H, Ar-Hs), 7.06 (ddd, *J* = 8.0, 6.9, 1.1 Hz, 1H, Ar-H), 6.75 – 6.73 (m, 2H, Ar-Hs). HRMS (ESI): *m*/*z*: [M + H]^+^ calcd. 543.1928 and found 543.1918. Anal. Calcd. (Found) For C_35_H_22_N_6_O: C, 77.48 (77.29); H, 4.09 (4.10); N, 15.49 (15.55)%.

#### 4-(2-Hydroxy-3-methoxyphenyl)-3,6-di (1H-indol-3-yl)-1-phenyl-1H-pyrazolo [3,4-b]pyridine-5-carbonitrile (7i)

Yield (88%) as a yellow powder, with Mp: >300 °C. ^1^H NMR (500 MHz, DMSO*d*_6_) δ (ppm): 12.17 (s, 1H, OH), 11.65 (s, 1H, NH), 11.36 (d, *J* = 2.6 Hz, 1H, NH), 8.35 (d, *J* = 3.1 Hz, 1H, Ar-H), 8.24 – 8.22 (m, 1H, Ar-H), 8.12 – 8.10 (m, 1H, Ar-H), 7.84 (d, *J* = 2.6 Hz, 1H, Ar-H), 7.73 – 7.71 (m, 2H, Ar-Hs), 7.57 – 7.53 (m, 2H, Ar-Hs), 7.48 – 7.46 (m, 1H, Ar-H), 7.41 (ddd, *J* = 9.0, 7.3, 1.2 Hz, 2H, Ar-Hs), 7.26 (dd, *J* = 8.0, 1.4 Hz, 1H, Ar-H), 7.15 – 7.08 (m, 5H, Ar-Hs), 6.89 (t, *J* = 7.9 Hz, 1H, Ar-H), 3.76 (s, 3H, OCH_3_). HRMS (ESI): *m*/*z*: [M + H]^+^ calcd. 573.2034 and found 573.2026. Anal. Calcd. (Found) For C_36_H_24_N_6_O_2_: C, 75.51 (75.69); H, 4.22 (4.23); N, 14.68 (14.73)%.

#### 4-(2,5-Dimethoxyphenyl)-3,6-di (1H-indol-3-yl)-1-phenyl-1H-pyrazolo [3,4-b]pyridine-5-carbonitrile (7j)

Yield (90%) as a yellow powder, with Mp: >300 °C. ^1^H NMR (700 MHz, DMSO*d*_6_) δ (ppm): 11.67 (d, *J* = 2.8 Hz, 1H, NH), 11.31 (d, *J* = 2.7 Hz, 1H, NH), 8.33 (d, *J* = 7.9 Hz, 1H, Ar-H), 7.86 – 7.85 (m, 2H, Ar-Hs), 7.81 (d, *J* = 2.8 Hz, 1H, Ar-H), 7.63 – 7.58 (m, 3H, Ar-Hs), 7.49 (dt, *J* = 8.2, 0.9 Hz, 1H, Ar-H), 7.42 – 7.39 (m, 2H, Ar-Hs), 7.35 (dt, *J* = 8.2, 1.0 Hz, 1H, Ar-H), 7.19 (ddd, *J* = 8.1, 7.0, 1.2 Hz, 1H, Ar-H), 7.14 (dddd, *J* = 10.4, 8.1, 6.9, 1.2 Hz, 2H, Ar-Hs), 7.07 (ddd, *J* = 7.9, 6.9, 1.0 Hz, 1H, Ar-H), 7.03 (d, *J* = 8.7 Hz, 1H, Ar-H), 6.79 – 6.77 (m, 2H, Ar-Hs), 3.90 (s, 3H, OCH_3_), 3.61 (s, 3H, OCH_3_). ^13^C NMR (176 MHz, DMSO*d*_6_) δ (ppm): 172.48, 153.97, 150.53, 145.02, 144.91, 139.32, 138.64, 136.42, 136.31, 135.50, 129.76, 128.37, 126.90, 125.91, 125.58, 124.45, 123.12, 122.36, 122.28, 122.19, 122.17, 120.36, 120.25, 119.93, 116.86, 113.01, 112.51, 112.16, 111.71, 109.08, 108.37, 99.97, 82.96, 56.97, 55.55. HRMS (ESI): *m*/*z*: [M + H]^+^ calcd. 587.2190 and found 587.2193. Anal. Calcd. (Found) For C_37_H_26_N_6_O_2_: C, 75.75 (75.97); H, 4.47 (4.44); N, 14.33 (14.39)%.

#### 4,6-Di (1H-indol-3-yl)-1,3-diphenyl-1H-pyrazolo [3,4-b]pyridine-5-carbonitrile (10)

Yield (89%) as a yellow powder, with Mp: >300 °C. ^1^H NMR (700 MHz, DMSO*d*_6_) δ (ppm): 12.18 (s, 2H, NH), 8.37 (s, 4H, Ar-Hs), 8.14 – 8.13 (m, 4H, Ar-Hs), 7.51 (dt, *J* = 8.1, 0.9 Hz, 4H, Ar-Hs), 7.25 (dtd, *J* = 17.5, 7.1, 1.3 Hz, 8H, Ar-Hs). Anal. Calcd. (Found) For C_35_H_22_N_6_: C, 79.83 (80.06); H, 4.21 (4.21); N, 15.96 (16.04)%.

#### 3,4-Di (1H-indol-3-yl)-1,6-diphenyl-1H-pyrazolo [3,4-b]pyridine-5-carbonitrile (11)

Yield (80%) as a yellow powder, with Mp: >300 °C. ^1^H NMR (500 MHz, DMSO*d*_6_) δ (ppm): 11.79 (s, 2H, 2NH), 8.54 (q, *J* = 2.6 Hz, 2H, Ar-Hs), 8.42 (d, *J* = 7.6 Hz, 2H, Ar-Hs), 8.34 – 8.32 (m, 4H, Ar-Hs), 7.55 (d, *J* = 5.3 Hz, 6H, Ar-Hs), 7.48 – 7.46 (m, 2H, Ar-Hs), 7.19 (pd, *J* = 7.0, 1.3 Hz, 4H, Ar-Hs). Anal. Calcd. (Found) For C_35_H_22_N_6_: C, 79.83 (80.09); H, 4.21 (4.25); N, 15.96 (16.00)%.

#### 3,4,6-Tri (1H-indol-3-yl)-1-phenyl-1H-pyrazolo [3,4-b]pyridine-5-carbonitrile (12)

Yield (80%) as a yellow powder, with Mp: >300 °C. ^1^H NMR (500 MHz, DMSO*d*_6_) δ (ppm): 12.16 (s, 3H, 3NH), 8.34 (s, 4H, Ar-Hs), 8.14 – 8.09 (m, 4H, Ar-Hs), 7.49 – 7.45 (m, 4H, Ar-Hs), 7.20 (tt, *J* = 7.3, 5.6 Hz, 8H, Ar-Hs). Anal. Calcd. (Found) For C_37_H_23_N_7_: C, 78.57 (78.35); H, 4.10 (4.12); N, 17.33 (17.28)%.

### Biological evaluation

#### Cell lines

MV4-11 cancer cell line (purchased from the German Collection of Microorganisms) was cultivated in RPMI-1640 medium and, while K562 and G361 cell lines (obtained from European Collection of Cell Cultures) were grown in DMEM medium. All cell lines were cultivated according to the provider’s instructions in a humidified CO_2_ incubator at 37 °C, and both media were supplemented with 10% fetal bovine serum, 100 U/mL penicillin, 100 mg/mL streptomycin and glutamine (4 mM) (Eldehna et al., 2025; Shaldam et al., 2023).

#### Cell viability and drug combination assays

Cells were plated in 96-well plates and incubated overnight. Then, they were treated with increasing concentrations of tested compounds. After 72-h treatment, resazurin (Merck) was added to each well for 4 h. The fluorescence of resorufin, a reduced product corresponding to live cells, was measured at 544 nm (excitation) and 590 nm (emission) using a Fluoroskan Ascent microplate reader (Labsystems). GI_50_ values, defined as the compound concentration lethal to 50% of the cancer cells, were calculated from the obtained dose-response curves. Synergy heatmaps were generated using the Highest single agent (HSA) model in Synergy Finder (3.0) (Zheng et al., 2022).

#### Flow cytometry

MV4-11 asynchronously growing cells were treated with test compounds at different doses of tested compounds for 24 h. Treated cells were then harvested and fixed with ice-cold 70% ethanol. For the cell cycle analysis, fixed cells were washed with PBS and stained with propidium iodide. After 30 min of staining, the distribution of cells in the cell cycle was analyzed using flow cytometry with a 488 nm laser (BD FACS Verse), and the sub-G1 population was quantified in BD FACSuite™ software (version 1.0.6.).

#### Immunoblotting and antibodies

Cell lysates were extracted by RIPA buffer and then separated by SDS-polyacrylamide gels and electrotransfered onto nitrocellulose membranes. After 1 h of blocking, membranes were incubated with specific primary antibodies overnight at 4 °C. Then they were washed and incubated for 1 h with peroxidase-conjugated secondary antibodies. Peroxidase activity was detected using SuperSignal West Pico reagents (Thermo Scientific) and LAS-4000 CCD camera (Fujifilm). Specific primary and secondary antibodies were purchased from Cell Signaling Technology (peroxidase-conjugated secondary antibodies; anti-PARP, clone 46D11; anti-Mcl-1, clone D35A5; anti-Caspase 7; anti-Caspase 9; anti-phospho-p53-Ser15; anti-CHK1, clone 2G1D5; anti-phospho-CHK1-Ser317; anti-cyclin E, clone HE12; anti-CDK2, clone 78B2), Santa Cruz Biotechnology (anti-β-actin, clone C4) and Merck (anti-phospho-histone H2AX-Ser139). Antibody anti-p53, clone DO-1, was kindly gifted by Dr. B. Vojtěšek (Masaryk Memorial Cancer Institute, Brno, Czech Republic).

#### Topoisomerase relaxation assay

The topoisomerase relaxation assays were performed with topoisomerase I or IIα enzymes (Inspiralis) according to manufacturer instructions. Topoisomerase I reaction was performed in assay buffer (20 mM Tris-HCl pH 7.5, 200 mM NaCl, 0.25 mM EDTA, 5% glycerol, 50 μg/μL albumin) containing 0.5 µg supercoiled pBR322 in reaction volume of 30 μL at 37 °C and 350 rpm for 30 min. Similarly, the topoisomerase II reaction was performed in the same incubation conditions in the presence of 0.5 µg supercoiled pBR322. The assay buffer (50 mM Tris-HCl pH 7.5, 125 mM NaCl, 10 mM MgCl2, 5 mM DTT, 100 μg/μL albumin) was supplemented by 1 mM ATP. Both types of reactions were stopped by adding 30 µL of GSTEB (8% (w/v) glycerol, 25 mM Tris-HCl pH 8.0, 2 mM EDTA, 0.1 mg/mL bromphenol blue) and 30 µL of chloroform/isoamyl alcohol (v:v, 24:1). The reaction products were separated by 5% agarose gel electrophoresis and visualized using GelRed nucleic acid stain (Biotium). The relaxation level of pBR322 was visualized by an FLA-7000 digital image analyzer (FujiFilm).

### Molecular docking

The coordinates of TOPI (1K4T (Staker et al., 2002)) and TOPII (PDB: 3QX3 (Wu et al., 2011)) were obtained from the RCSB-PDB site. As part of the docking study, the three-dimensional structures of 7b, 7e and the cocrystal ligand were sketched and refined using Marvin Sketch (Ryan and Wedge, 2005). Docking activities were performed using AutoDock Vina (Trot et al., 2010). The coordinates of the active site (x, y, z) were 21.5/-3.9/28.3 with size 16.0/23.8/13.8 for TOPI and 32.9/95.4/50.8 with size 24.5/20.2/17.5 for TOPII. The Discovery Studio 2021 client was used to construct the 3D visualization and 2D schematic presentation (Eliwa et al., 2021).

## Data Availability

The original contributions presented in the study are included in the article/Supplementary Material, further inquiries can be directed to the corresponding authors.
