# Correction: Discovery of potent bisindole-based pyrazolopyridine derivatives as topoisomerase inhibitors: DNA damage induction and synergistic antileukemic activity

**DOI:** 10.3389/fphar.2026.1895851

**Published:** 2026-06-15

**Authors:** Wagdy M. Eldehna, Haytham O. Tawfik, Denisa Veselá, Miroslav Peřina, Ahmed T. Negmeldin, Zainab M. Elsayed, Taghreed A. Majrashi, Veronika Vojáčková, Mostafa M. Elbadawi, Moataz A. Shaldam, Vladimír Kryštof, Hatem A. Abdel-Aziz

**Affiliations:** 1 Department of Pharmaceutical Chemistry, Faculty of Pharmacy, Kafrelsheikh University, Kafrelsheikh, Egypt; 2 Department of Pharmaceutical Chemistry, Faculty of Pharmacy, Tanta University, Tanta, Egypt; 3 Department of Experimental Biology, Faculty of Science, Palacký University Olomouc, Olomouc, Czechia; 4 Department of Pharmaceutical Sciences, College of Pharmacy and Thumbay Research Institute for Precision Medicine, Gulf Medical University, Ajman, United Arab Emirates; 5 Department of Pharmaceutical Organic Chemistry, Faculty of Pharmacy, Cairo University, Cairo, Egypt; 6 Scientific Research and Innovation Support Unit, Faculty of Pharmacy, Kafrelsheikh University, Kafr El-Shaikh, Egypt; 7 Department of Pharmacognosy, College of Pharmacy, King Khalid University, Abha, Saudi Arabia; 8 Applied Organic Chemistry Department, National Research Center, Cairo, Egypt

**Keywords:** bis-indole, Leukemia, molecular modeling, synergistic therapy, topoisomerase inhibitors

In the published article, there was an error in the Funding statement. The correct Funding statement reads:

The author(s) declared that financial support was received for this work and/or its publication. The authors extend their appreciation to the Deanship of Research and Graduate Studies at King Khalid University for funding this work through Large Research Project under grant number (RGP2/500/46). The author(s) declared that financial support was received for this work and/or its publication. The study was supported by The European Union-Next Generation EU (The project National Institute for Cancer Research, Programme EXCELES, ID No. LX22NPO5102).

The original article has been updated.

