# Supplementary material for: Discovery of potent bisindole-based pyrazolopyridine derivatives as topoisomerase inhibitors: DNA damage induction and synergistic antileukemic activity
Source: Front Pharmacol. 2026 Mar 4;17:1745220. doi: 10.3389/fphar.2026.1745220 (PMC12996152; doi:10.3389/fphar.2026.1745220)
Supplement: Supplementary file 1 [file DataSheet1.pdf]

Supporting Information

Discovery of Potent Bisindole-based Pyrazolopyridine Derivatives as Topoisomerase Inhibitors: DNA Damage Induction and Synergistic Antileukemic Activity

Wagdy M. Eldehna [a,1,*], Haytham O. Tawfik [b,1,*], Denisa Veselá [c], Miroslav Peřina[c], Ahmed T. Negmeldin [d,e,*], Zainab M. Elsayed [f], Taghreed A Majrashi [g], Veronika Vojáčková [c], Mostafa M. Elbadawi [a], Moataz A. Shaldam [a], Vladimír Kryštof [c], Hatem A. Abdel-Aziz [h]

[a] *Department of Pharmaceutical Chemistry, Faculty of Pharmacy, Kafrelsheikh University, P.O. Box 33516, Kafrelsheikh, Egypt*

[b] *Department of Pharmaceutical Chemistry, Faculty of Pharmacy, Tanta University, Tanta, 31527, Egypt*

[c] *Department of Experimental Biology, Faculty of Science, Palacký University Olomouc, Šlechtitelů 27, 77900 Olomouc, Czech Republic*

[d] *Department of Pharmaceutical Sciences, College of Pharmacy and Thumbay Research Institute for Precision Medicine, Gulf Medical University, Ajman, United Arab Emirates*

[e] *Department of Pharmaceutical Organic Chemistry, Faculty of Pharmacy, Cairo University, Cairo, Egypt*

[f] *Scientific Research and Innovation Support Unit, Faculty of Pharmacy, Kafrelsheikh University, Kafrelsheikh, Egypt*

[g] *Department of Pharmacognosy, College of Pharmacy, King Khalid University, Asir, Saudi Arabia*

[h] *Applied Organic Chemistry Department, National Research Center, Dokki 12622, Cairo, Egypt*

*Corresponding author: W. M. Eldehna wagdy2000@gmail.com, and H. O. Tawfik haytham.omar.mahmoud@pharm.tanta.edu.eg and A. T. Negmeldin dr.ahmedthabet@gmu.ac.ae

[1] Both authors contributed equally to this work.

Supporting Information

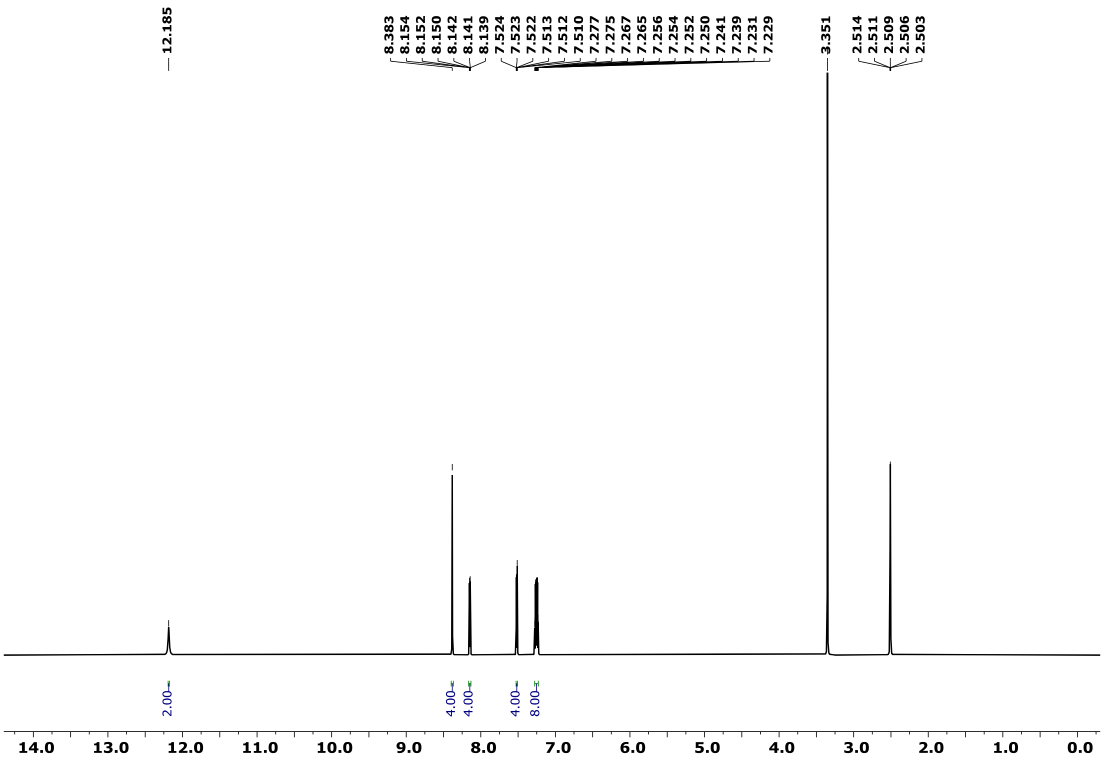

Figure S1. ^{1}H NMR (700 MHz, DMSO-d_6) spectrum of compound **7a**

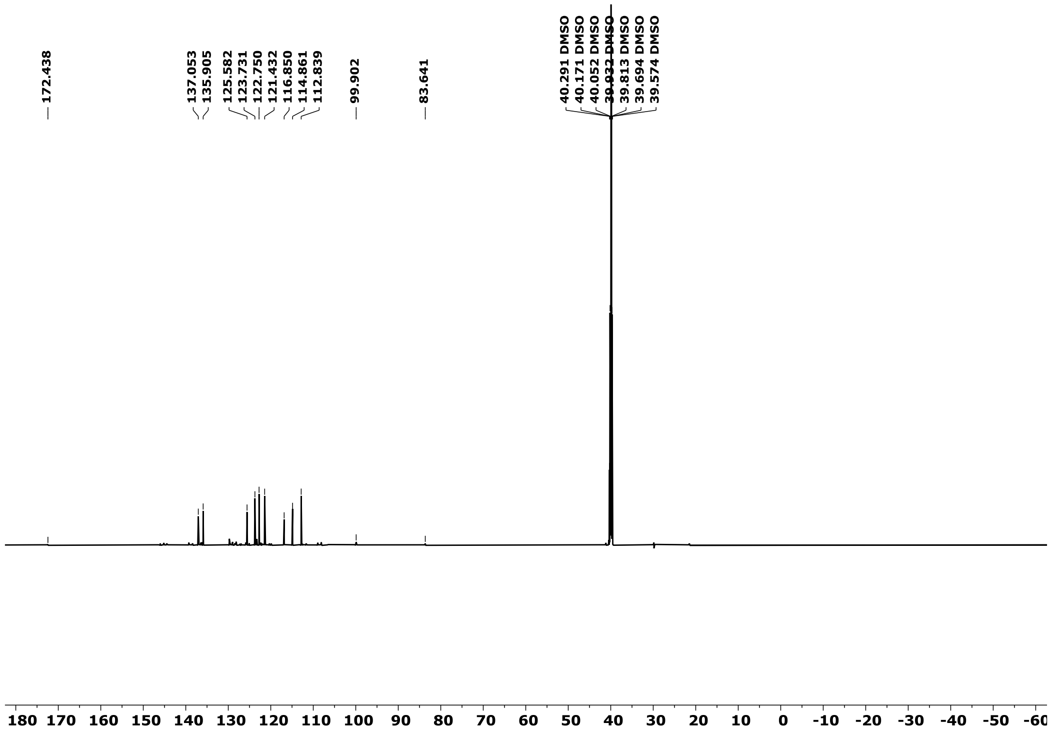

Figure S2. ^{13}C NMR (176 MHz, DMSO-d_6) spectrum of compound **7a**

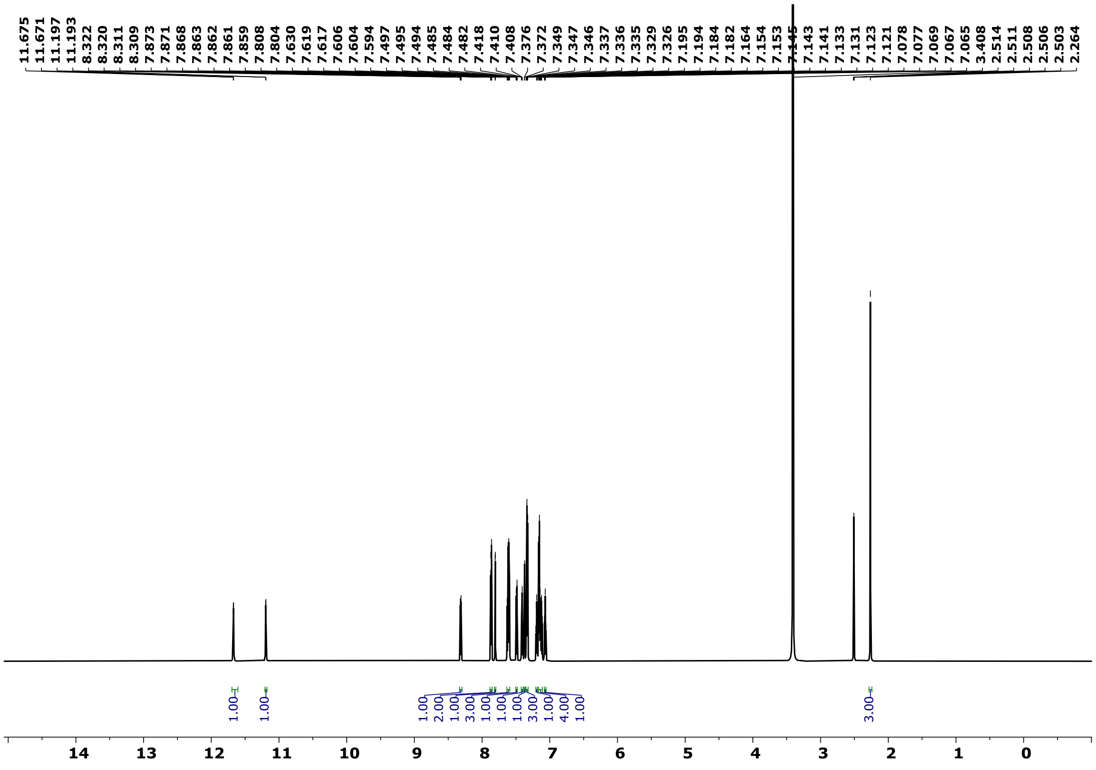

Figure S3. ^{1}H NMR (700 MHz, DMSO-d_6) spectrum of compound **7b**

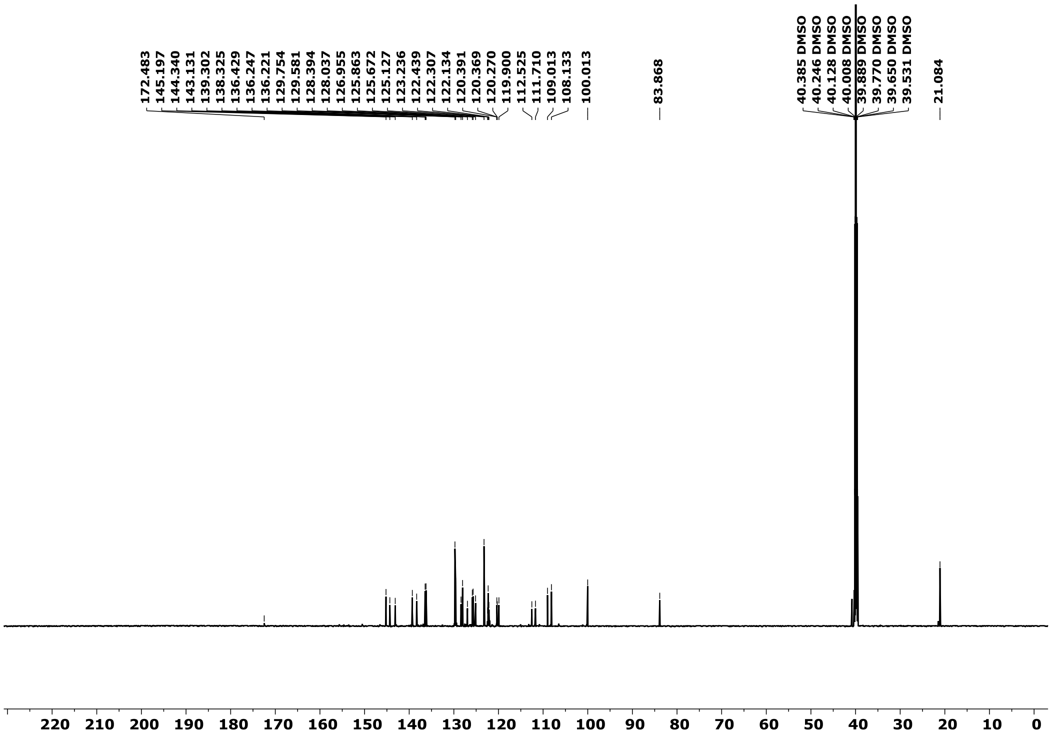

Figure S4. ^{13}C NMR (176 MHz, DMSO-d_6) spectrum of compound **7b**

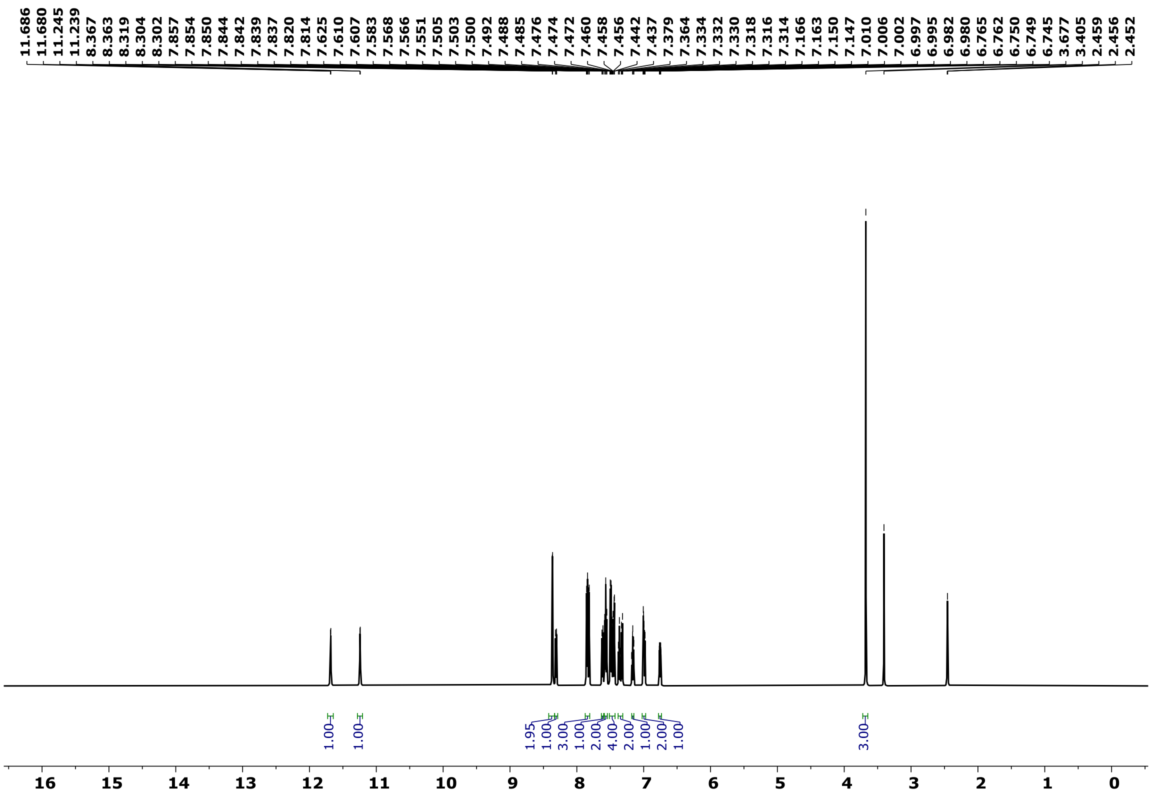

Figure S5. ^{1}H NMR (500 MHz, DMSO-d_6) spectrum of compound **7c**

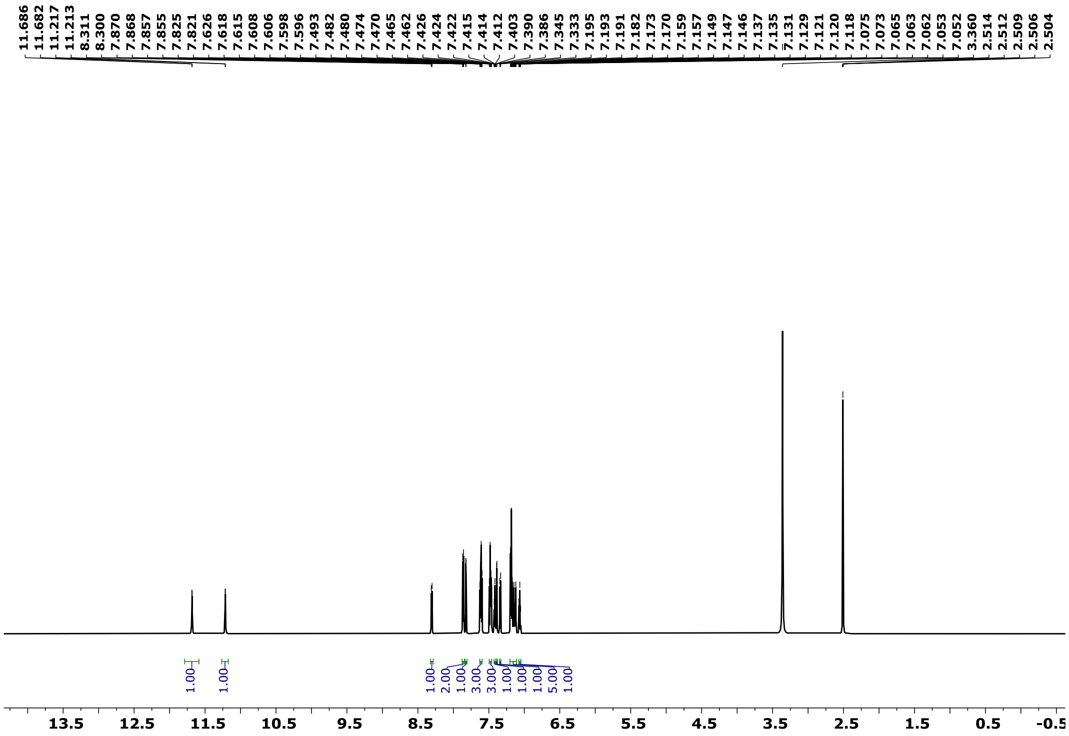

Figure S6. ^{1}H NMR (700 MHz, DMSO-d_6) spectrum of compound **7d**

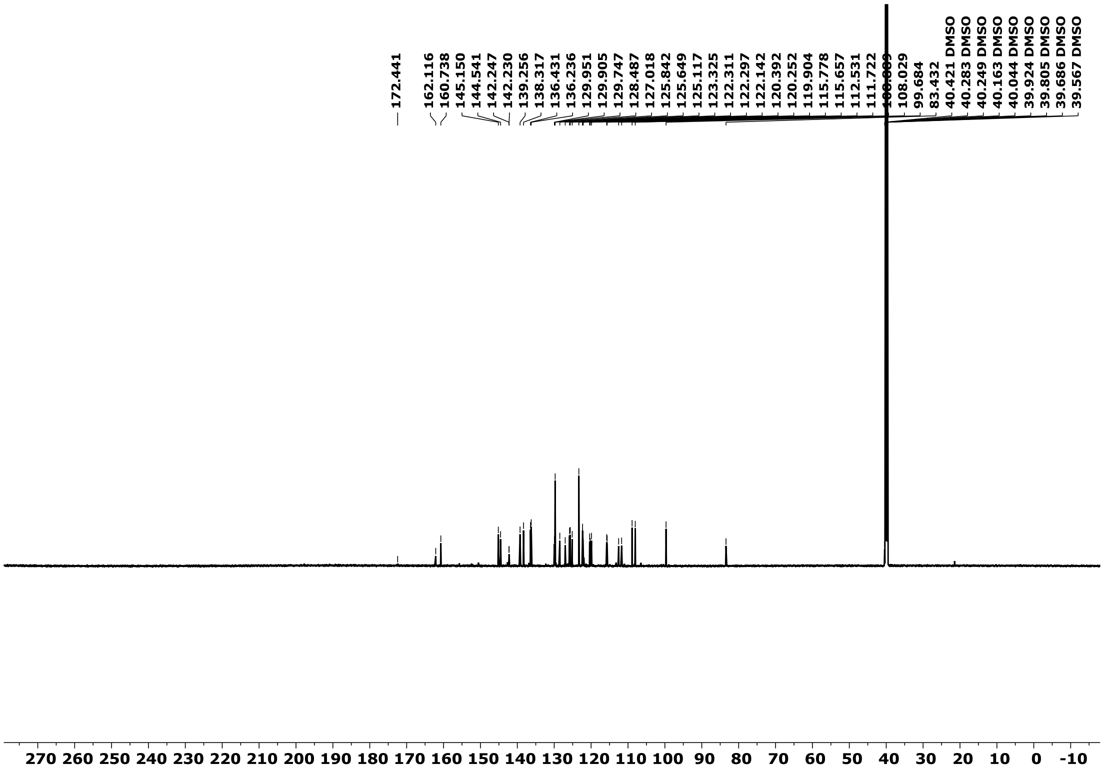

Figure S7. ^{13}C NMR (176 MHz, DMSO-d_6) spectrum of compound **7d**

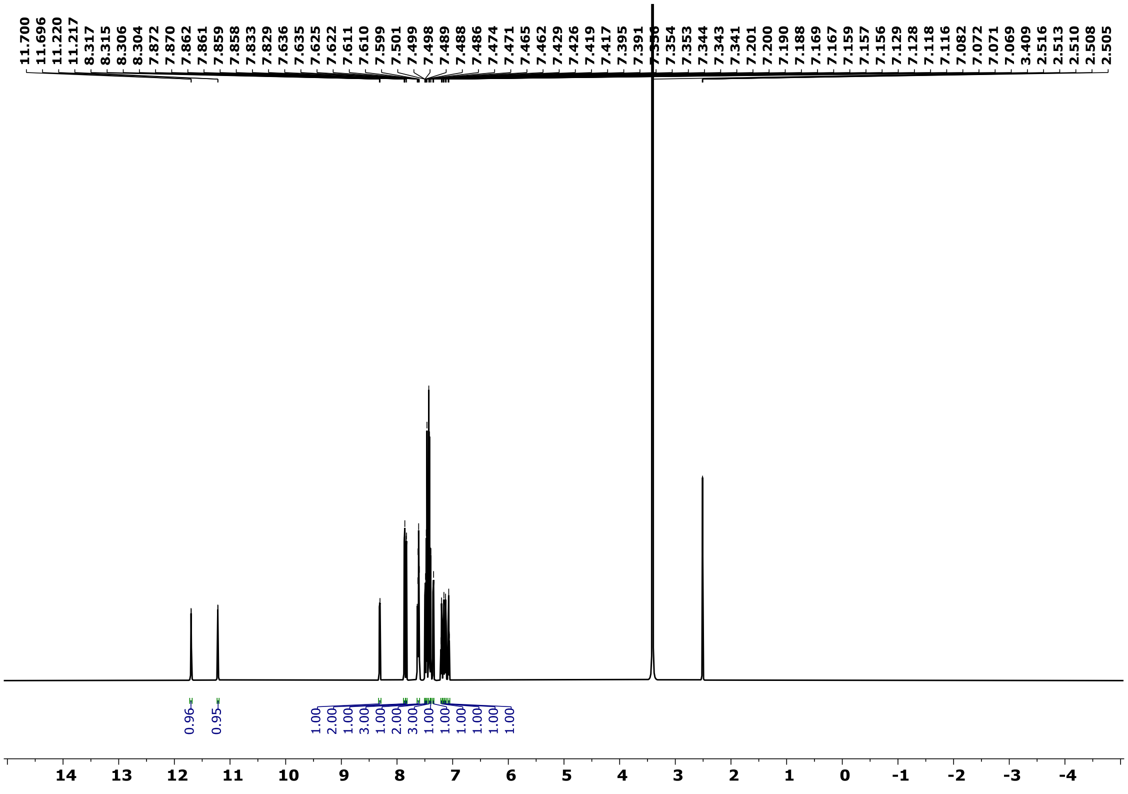

Figure S8. ^{1}H NMR (700 MHz, DMSO-d_6) spectrum of compound **7e**

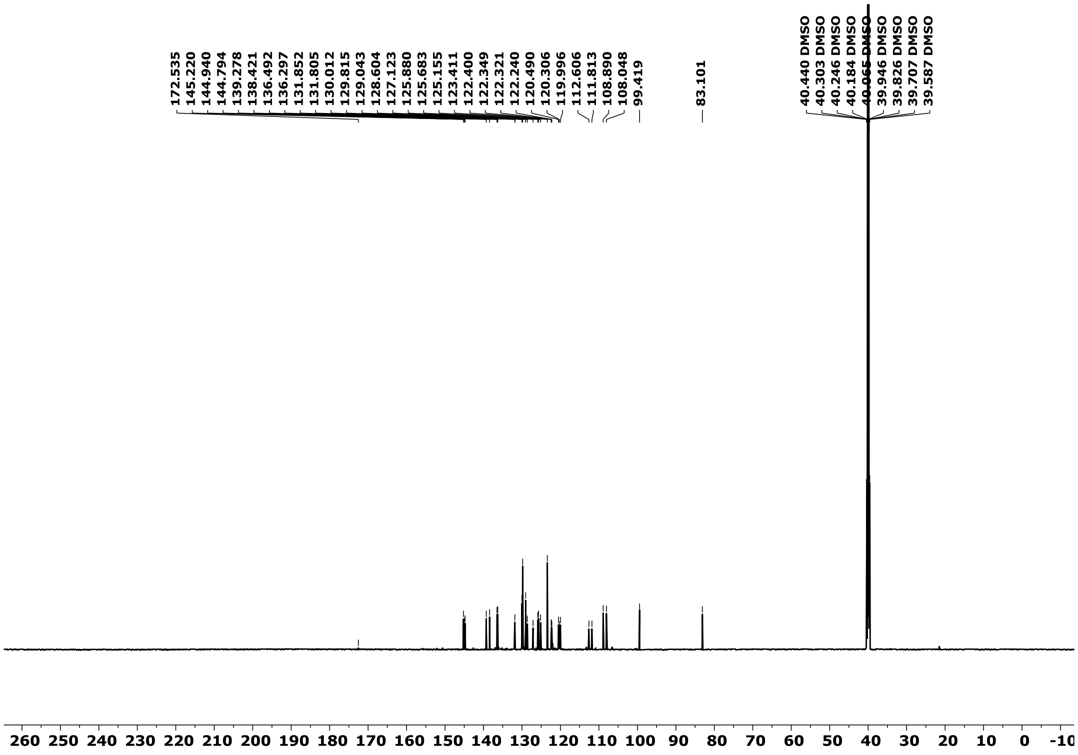

Figure S9. ^{13}C NMR (176 MHz, DMSO-d_6) spectrum of compound **7e**

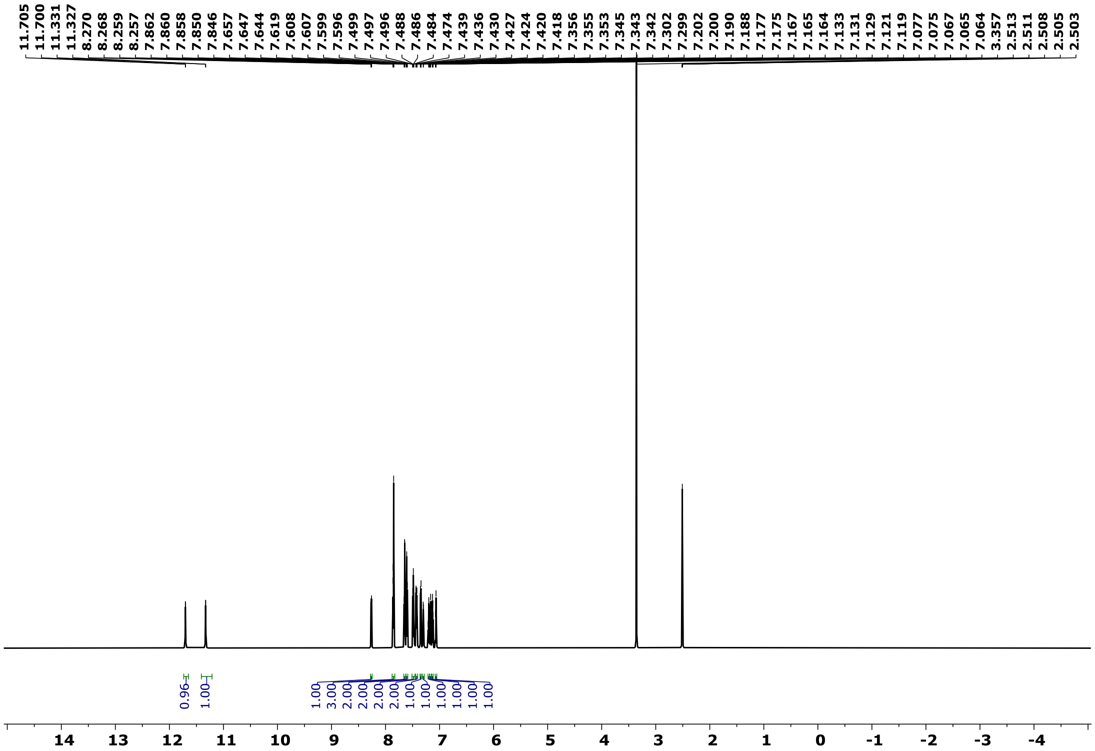

Figure S10. ^{1}H NMR (700 MHz, DMSO-d_6) spectrum of compound **7f**

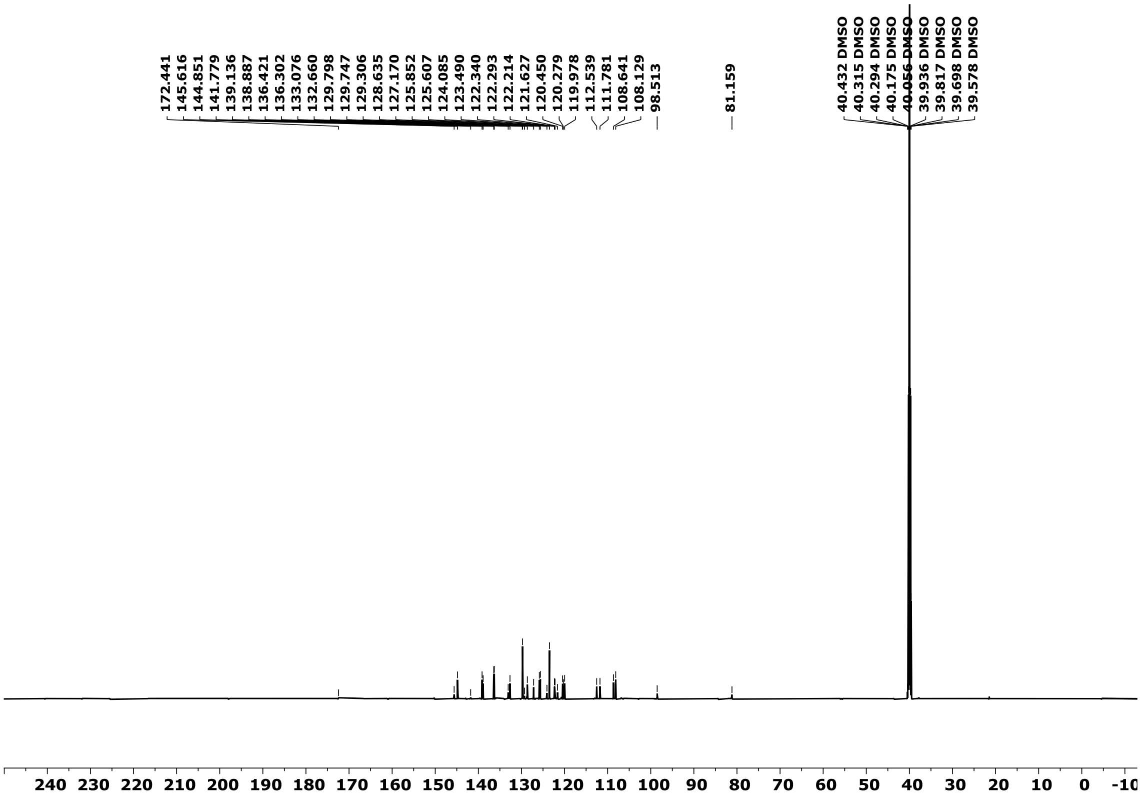

Figure S11. ^{13}C NMR (176 MHz, DMSO-d_6) spectrum of compound **7f**

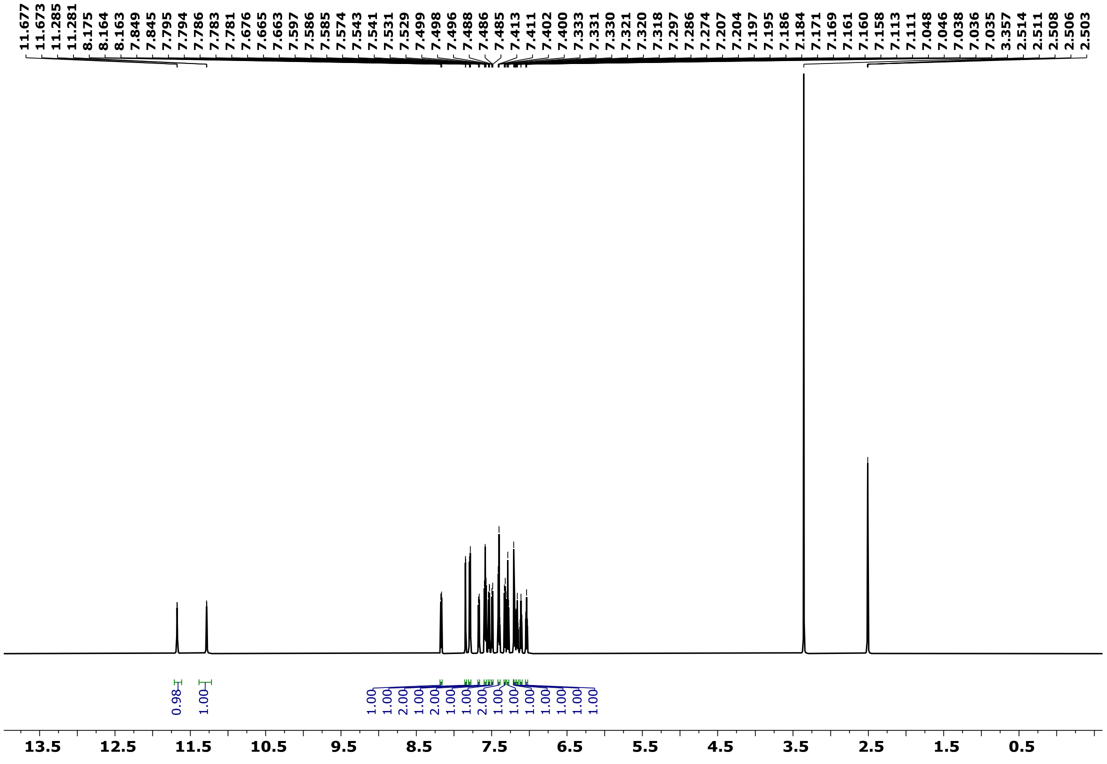

Figure S12. ^{1}H NMR (700 MHz, DMSO-d_6) spectrum of compound **7g**

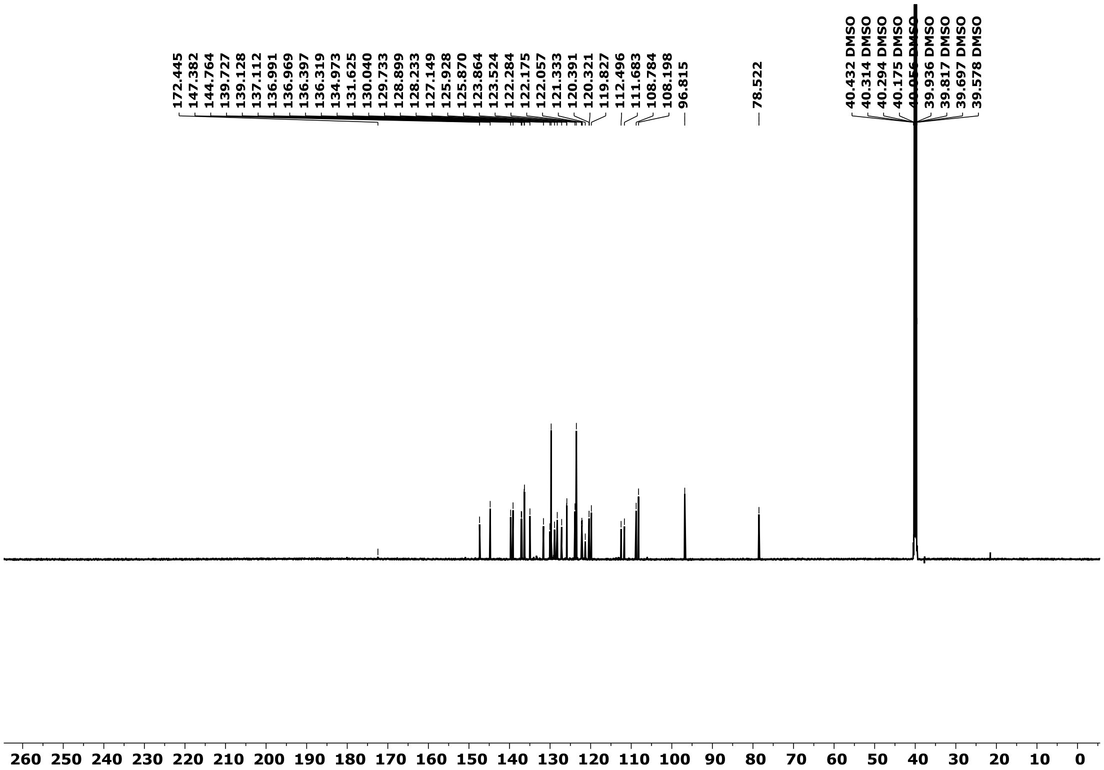

Figure S13. ^{13}C NMR (176 MHz, DMSO-d_6) spectrum of compound **7g**

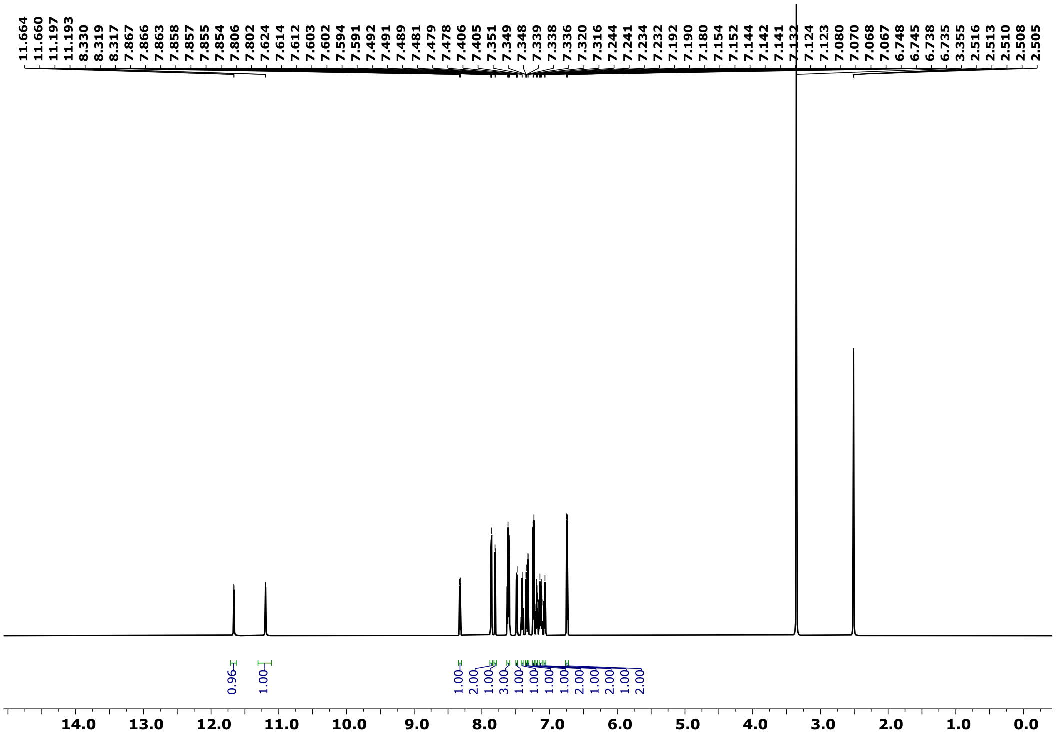

Figure S14. ^{1}H NMR (700 MHz, DMSO-d_6) spectrum of compound **7h**

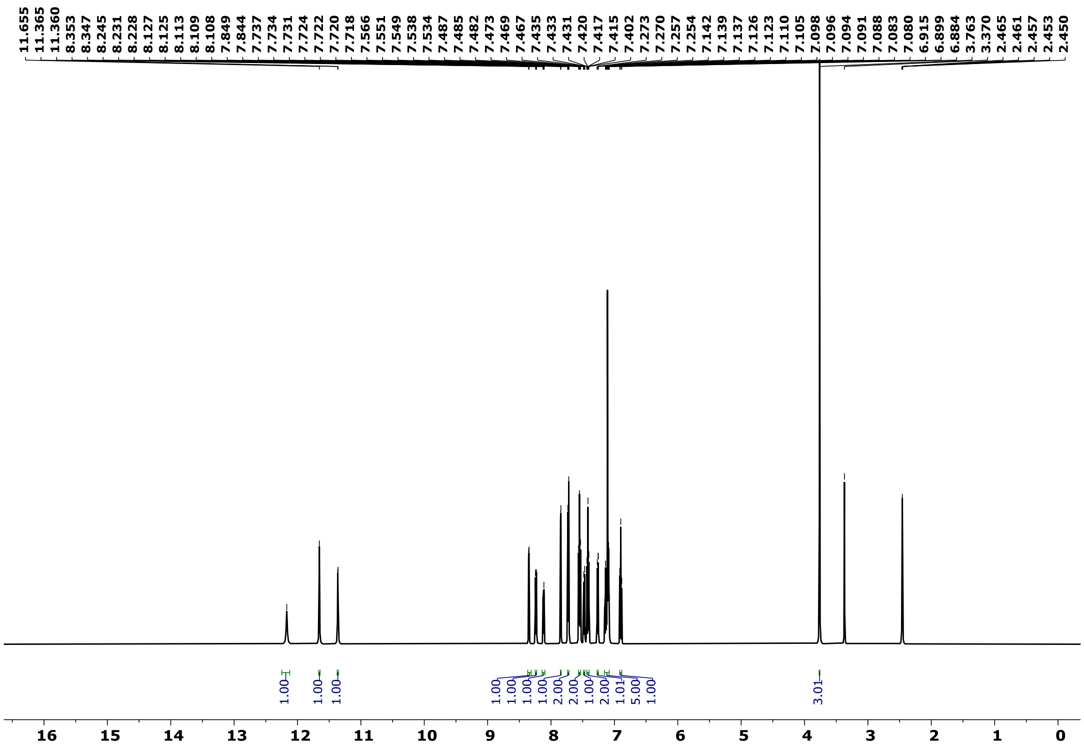

Figure S15. ^{1}H NMR (500 MHz, DMSO-d_6) spectrum of compound **7i**

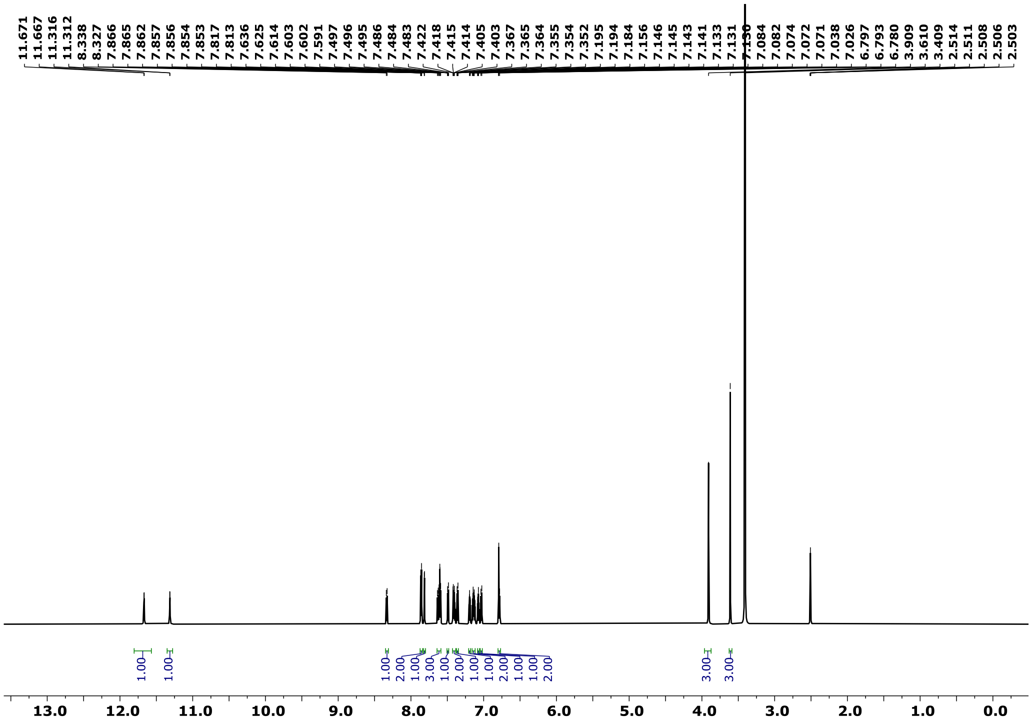

Figure S16. ^{1}H NMR (700 MHz, DMSO-d_6) spectrum of compound **7j**

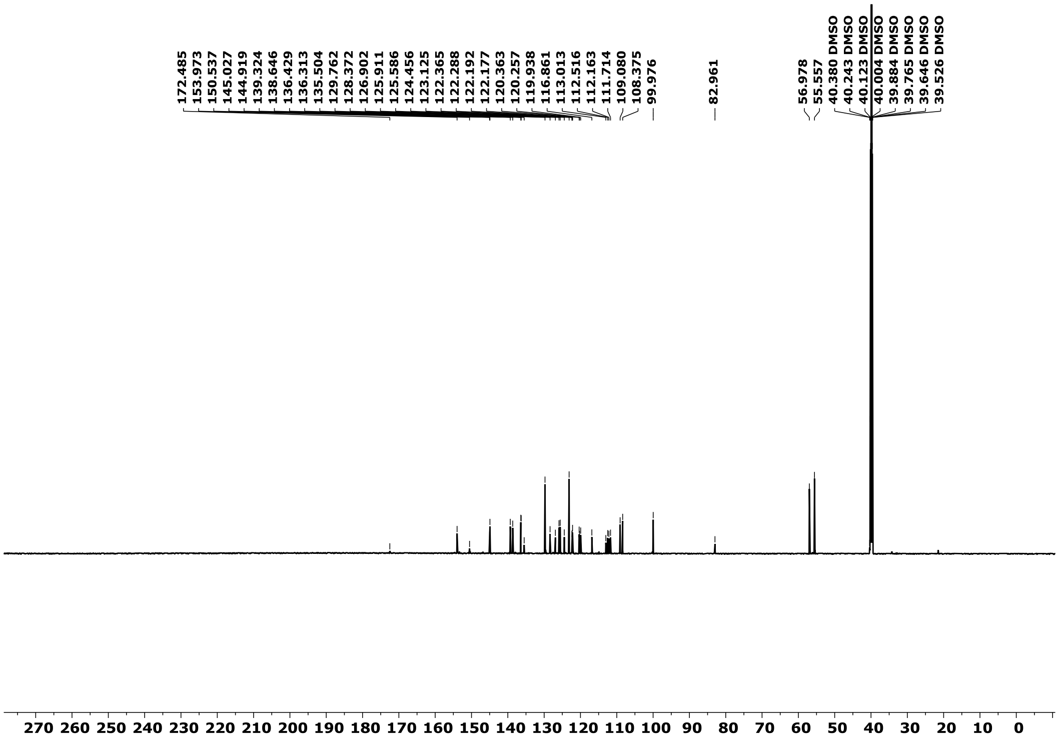

Figure S17. ^{13}C NMR (176 MHz, DMSO-d_6) spectrum of compound **7j**

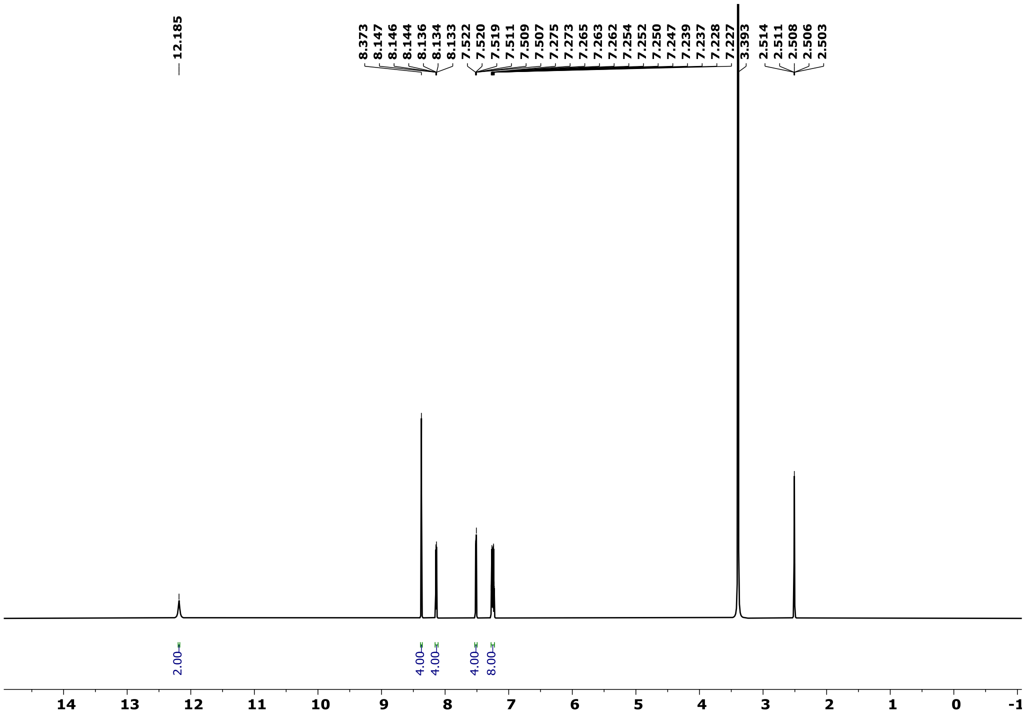

Figure S18. ^{1}H NMR (700 MHz, DMSO-d_6) spectrum of compound **10**

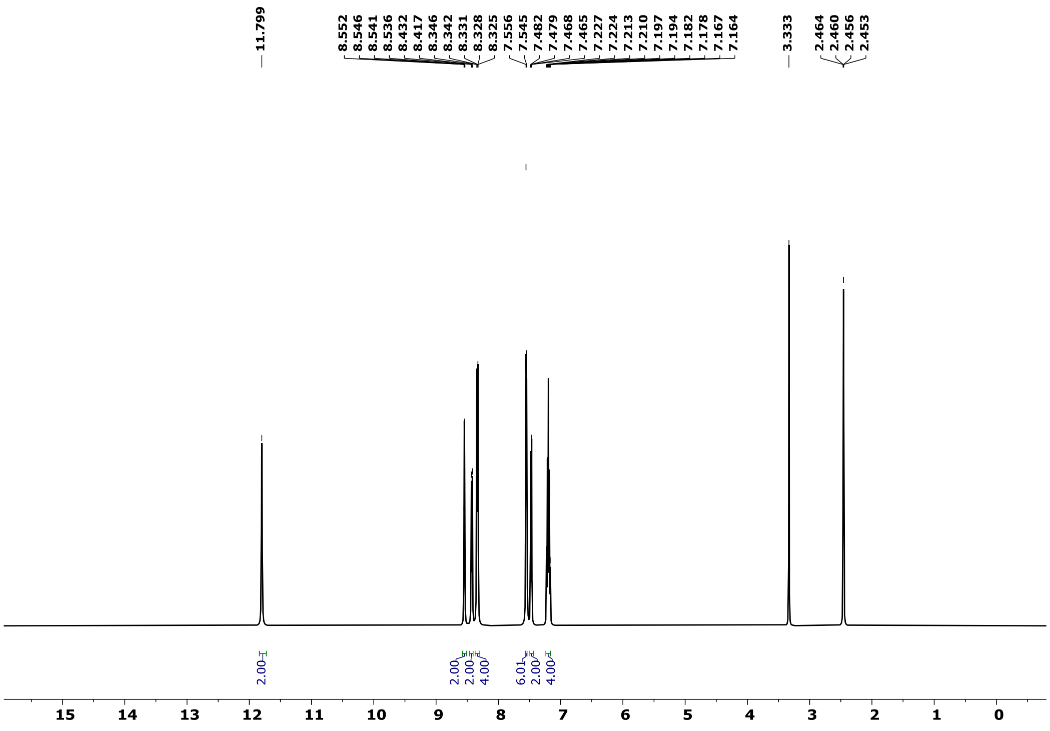

Figure S19. ^1H NMR (500 MHz, DMSO-d_6) spectrum of compound **11**

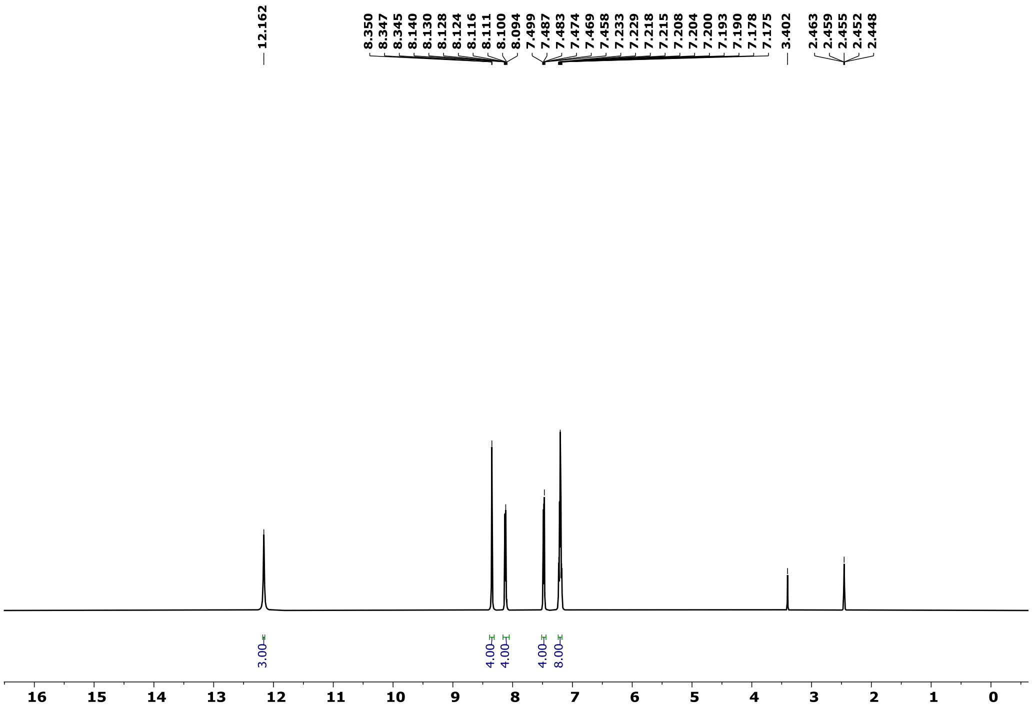

Figure S20. ^1H NMR (500 MHz, DMSO-d_6) spectrum of compound **12**

Supporting Information

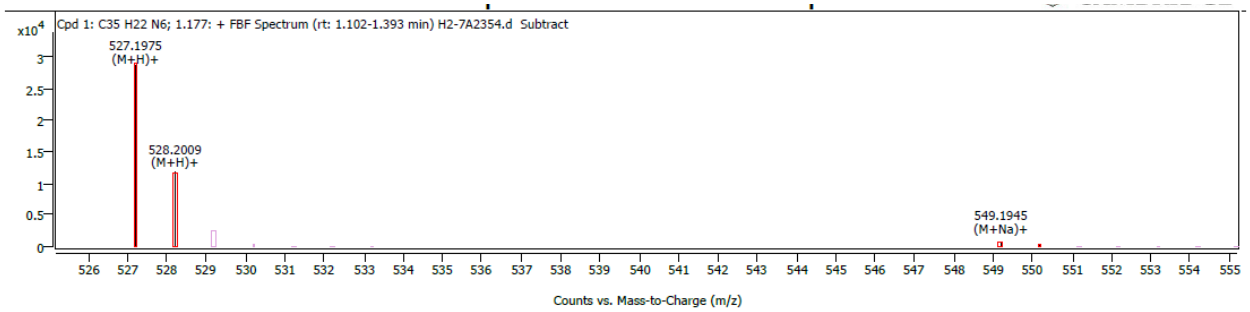

Figure S21. HRMS spectrum of compound **7a**

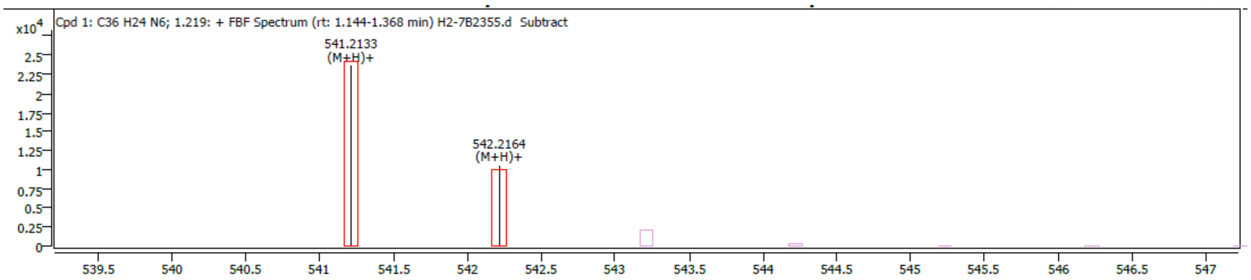

Figure S22. HRMS spectrum of compound **7b**

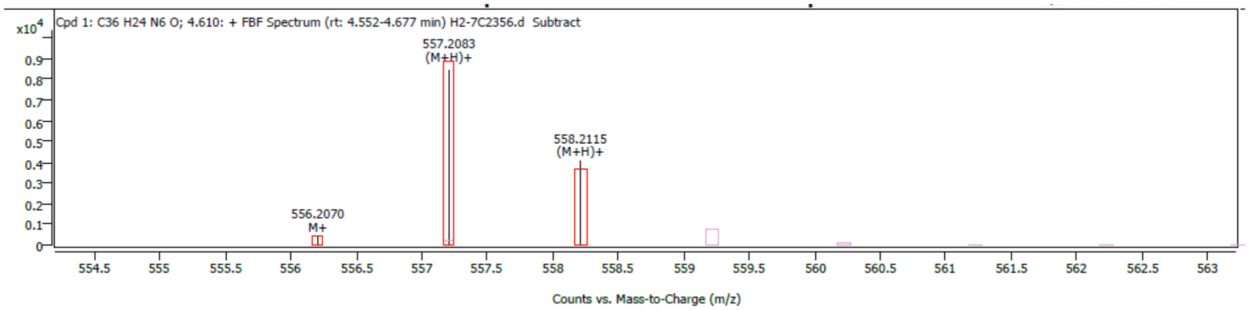

Figure S23. HRMS spectrum of compound **7c**

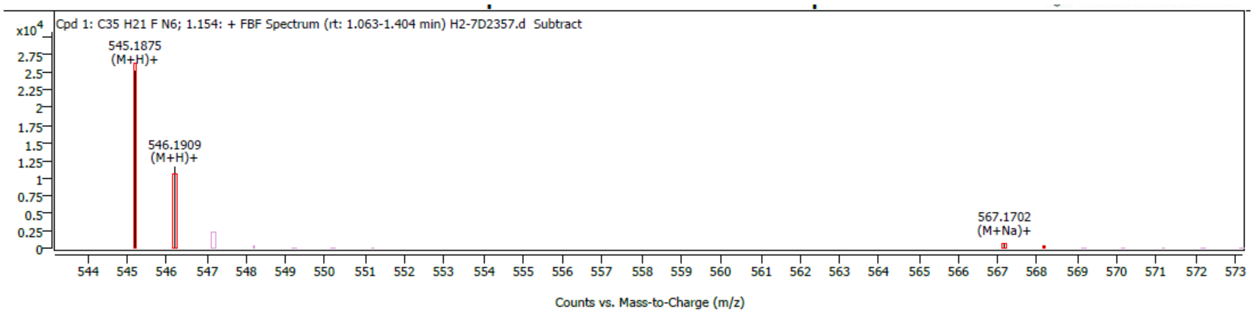

Figure S24. HRMS spectrum of compound **7d**

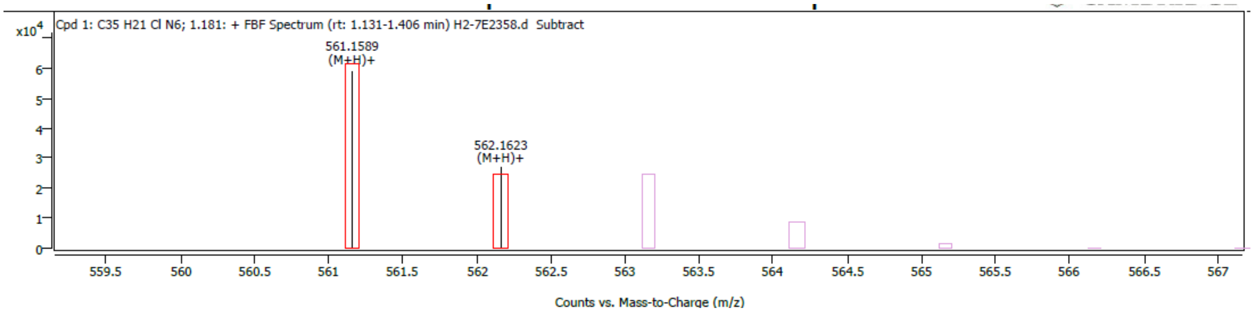

Figure S25. HRMS spectrum of compound **7e**

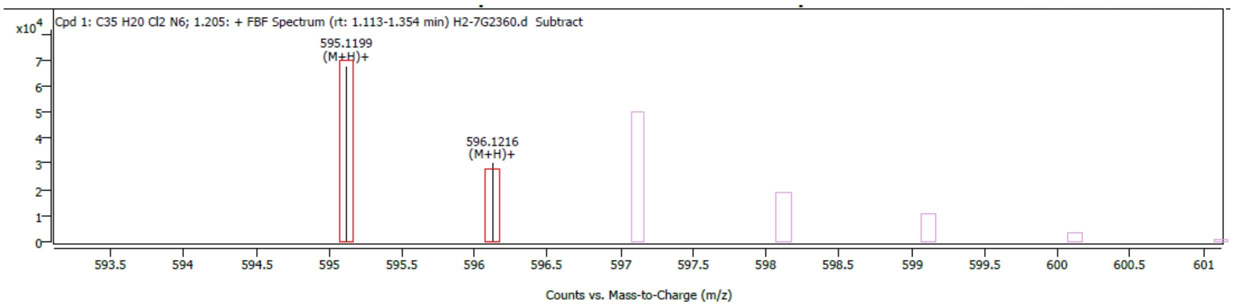

Figure S26. HRMS spectrum of compound **7g**

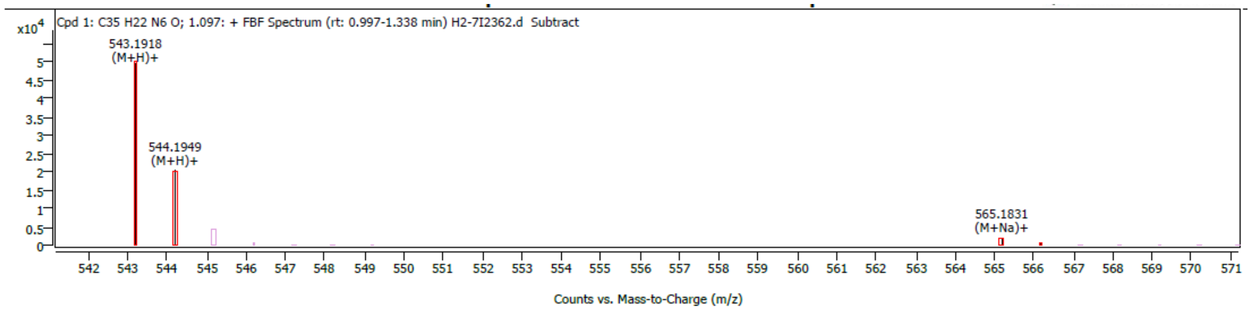

Figure S27. HRMS spectrum of compound **7h**

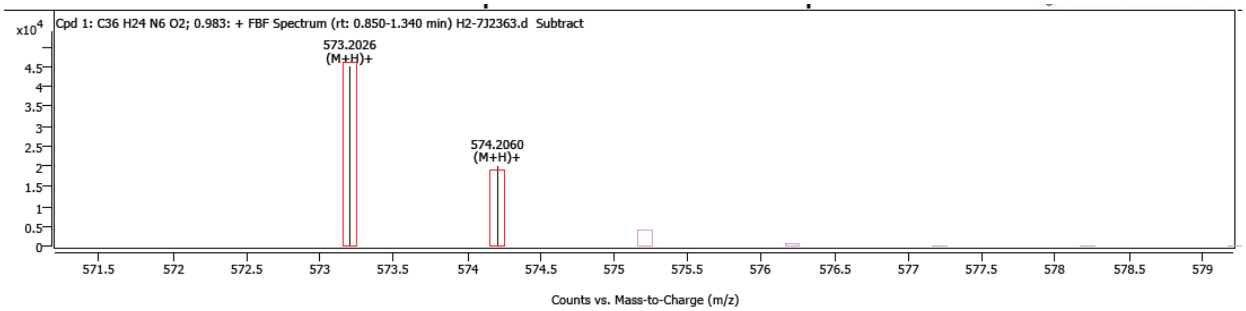

Figure S28. HRMS spectrum of compound **7i**

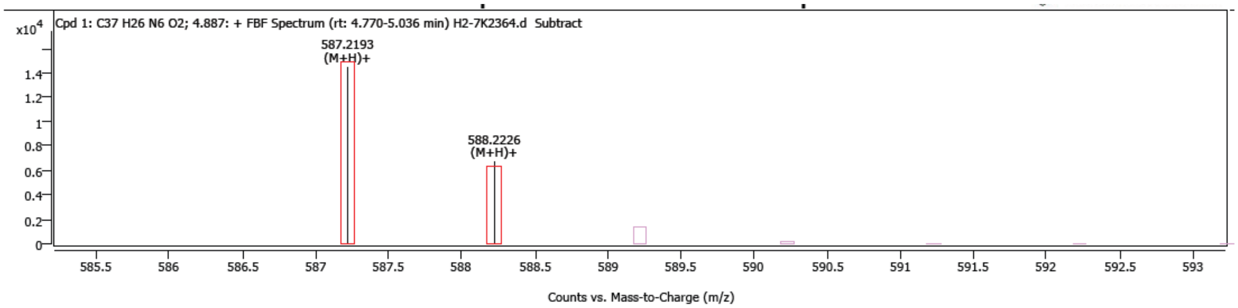

Figure S29. HRMS spectrum of compound **7j**

**Data file:**	C:\CHEM32\1\DATA\HAYTHAM\7B-005.D		
**Sample name:**	7b		
**Description:**	Solvent: DMSO + ACN, Flow rate: 1.5 mL/min<br>Mobile phase: 40% ACN + 60% Phosphate buffer		
**Sample amount:**	2.500	**Sample type:**	Sample
**Instrument:**	HPLC	**Location:**	Vial 1
**Injection date:**	6/8/2025 6:46:30 AM	**Injection:**	1 of 1
**Acq. method:**	HAYTHAM.M	**Injection volume:**	5.000
**Analysis method:**	HAYTHAM.M	**Acq. operator:**	SYSTEM
**Last changed:**	6/8/2025 7:01:53 AM<br>(modified after loading)		

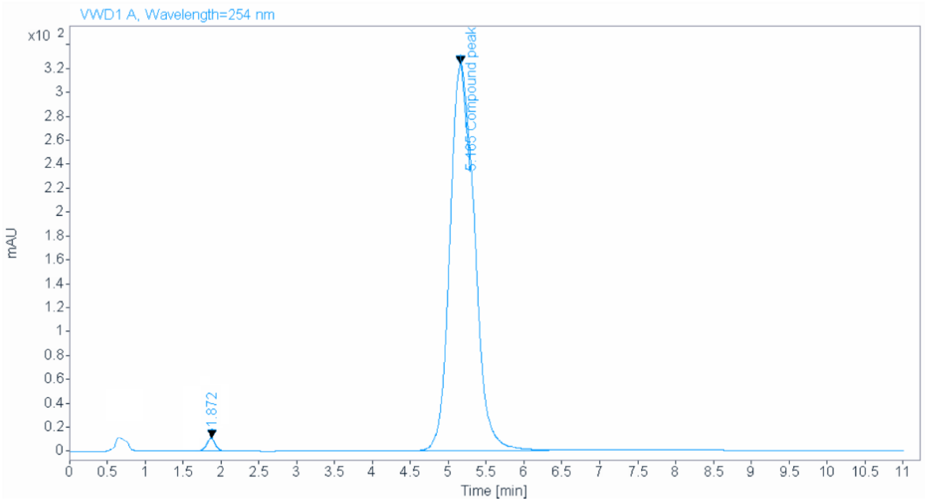

Signal: VWD1 A, Wavelength=254 nm

RT [min]	Type	Width [min]	Area	Height	Area%	Name
1.872	VB	0.0859	60.7488	10.3539	0.8119	
5.165	BV	0.3293	7421.9575	323.7516	99.1881	Compound peak
		Sum	7482.7063			

Figure S30. HPLC spectrum of compound **7b**

**Data file:**	C:\CHEM32\1\DATA\HAYTHAM\7D-002.D		
**Sample name:**	7d		
**Description:**	Solvent: DMSO + ACN, Flow rate: 1.5 mL/min<br>Mobile phase: 40% ACN + 60% Phosphate buffer		
**Sample amount:**	2.500	**Sample type:**	Sample
**Instrument:**	HPLC	**Location:**	Vial 2
**Injection date:**	6/8/2025 4:08:23 AM	**Injection:**	1 of 1
**Acq. method:**	HAYTHAM.M	**Injection volume:**	5.000
**Analysis method:**	HAYTHAM.M	**Acq. operator:**	SYSTEM
**Last changed:**	6/8/2025 4:34:15 AM<br>(modified after loading)		

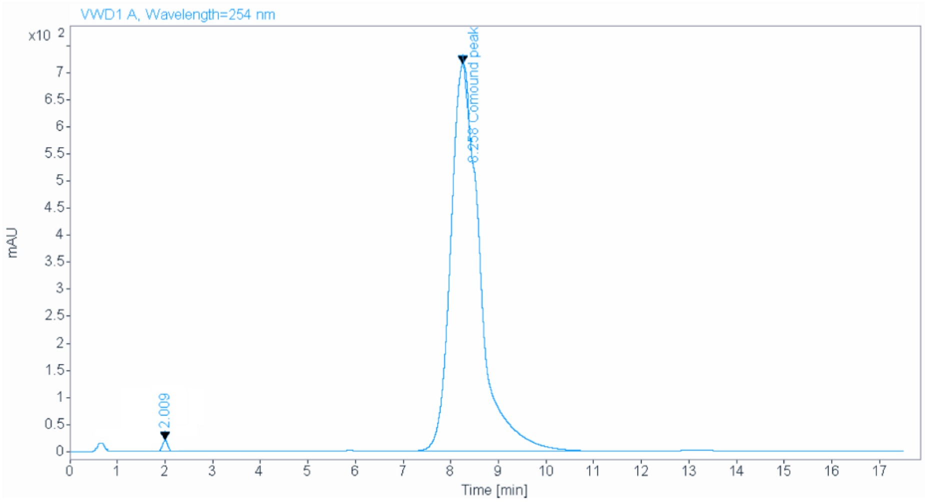

Signal: VWD1 A, Wavelength=254 nm

RT [min]	Type	Width [min]	Area	Height	Area%	Name
2.009	VB	0.0831	98.4541	18.1922	0.3223	
8.258	VB	0.6022	30451.0723	715.9852	99.6777	Comound peak
		Sum	30549.5264			

Figure S31. HPLC spectrum of compound **7d**

**Data file:**	C:\CHEM32\1\DATA\HAYTHAM\7E-001.D		
**Sample name:**	7e		
**Description:**	Solvent: DMSO + ACN, Flow rate: 1.5 mL/min<br>Mobile phase: 40% ACN + 60% Phosphate buffer		
**Sample amount:**	2.500	**Sample type:**	Sample
**Instrument:**	HPLC	**Location:**	Vial 3
**Injection date:**	6/8/2025 4:41:50 AM	**Injection:**	1 of 1
**Acq. method:**	HAYTHAM.M	**Injection volume:**	5.000
**Analysis method:**	HAYTHAM.M	**Acq. operator:**	SYSTEM
**Last changed:**	6/8/2025 4:51:17 AM<br>(modified after loading)		

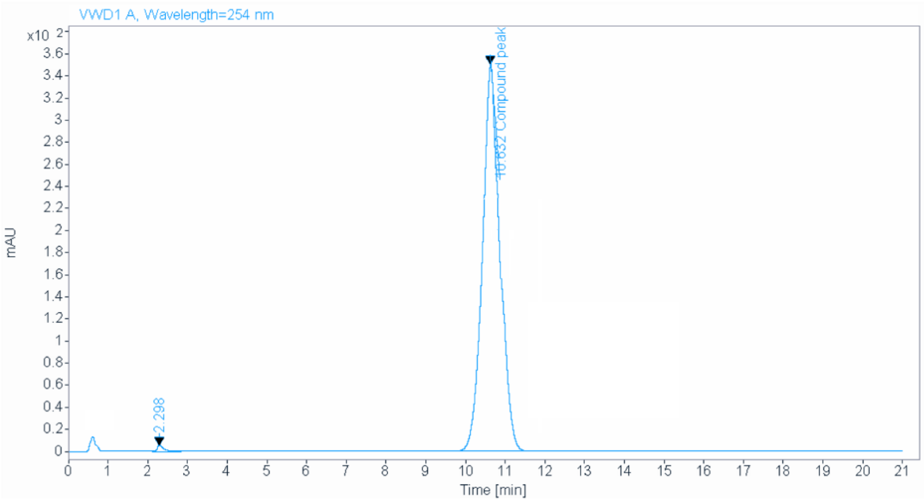

Signal: VWD1 A, Wavelength=254 nm

RT [min]	Type	Width [min]	Area	Height	Area%	Name
2.298	VV	0.1846	63.4913	5.2683	0.4475	
10.632	BV	0.5386	14125.2998	350.2096	99.5525	Compound peak
		Sum	14188.7911			

Figure S32. HPLC spectrum of compound **7e**

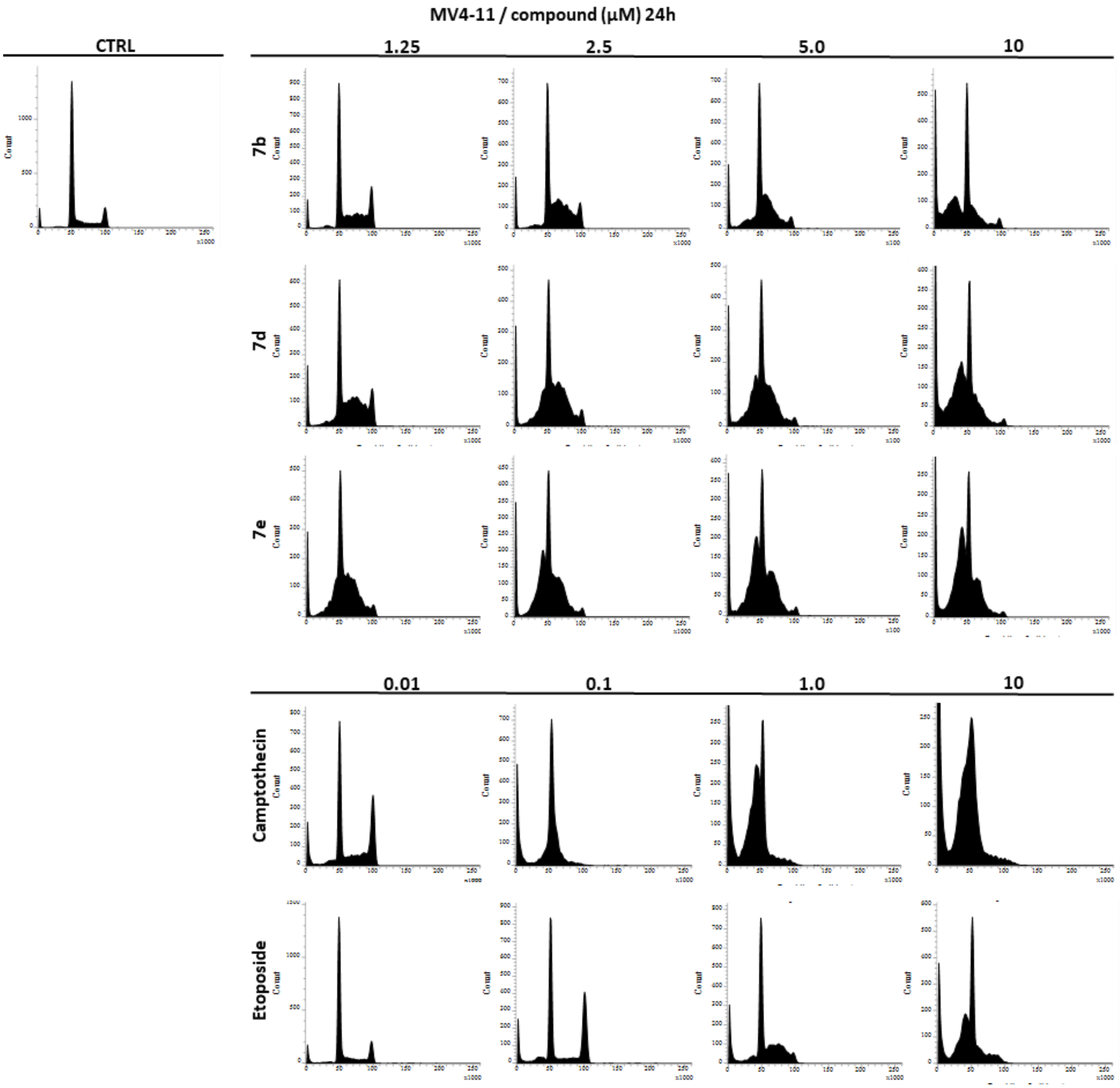

Figure S33. Histograms showing cell cycle distribution of MV4-11 treated for 24 hours with the indicated doses of **7b**, **7d** and **7e**, compared to camptothecin and etoposide.

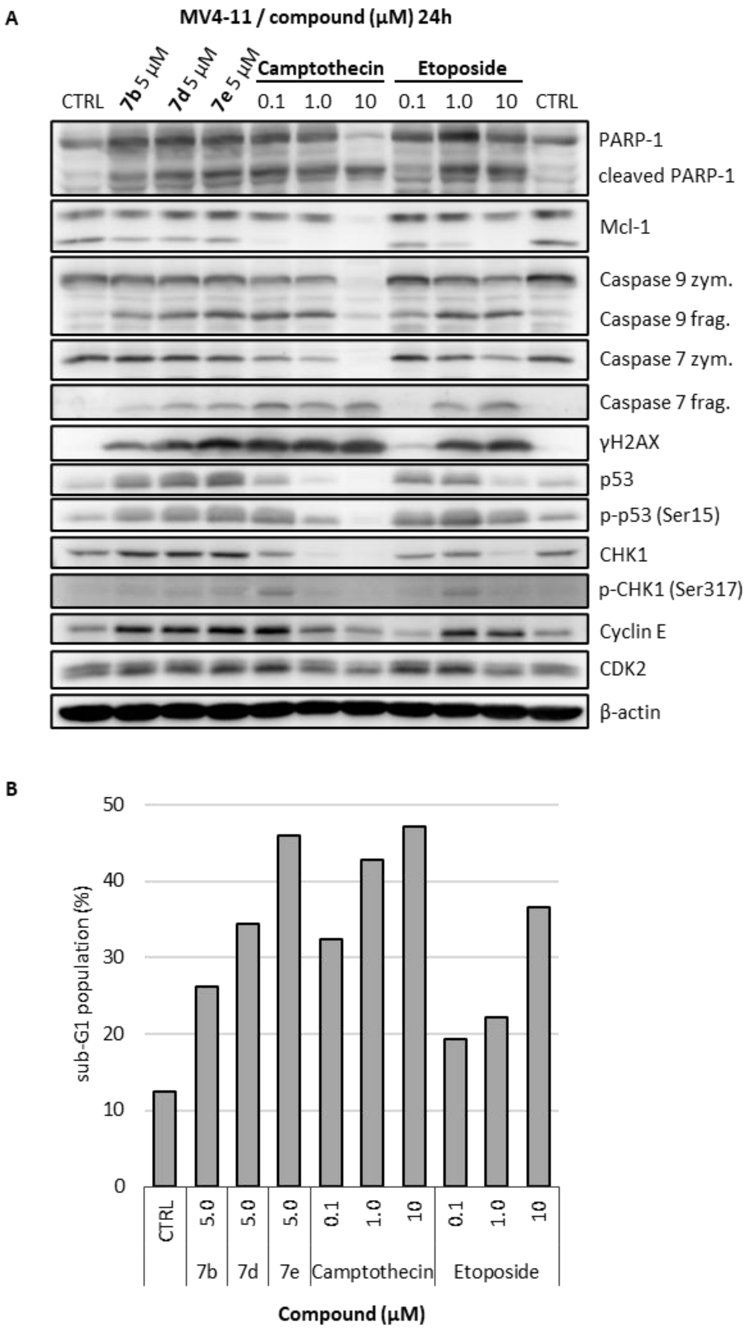

Figure S34. Comparison of the cellular effects of **7b**, **7d** and **7e** derivatives with camptothecin and etoposide in MV4-11 cells treated for 24 hours. (A) Immunoblotting of markers of cell death and DNA damage. β-actin detected as loading control. (B) Analysis of induction of cell death (sub-G1 population).

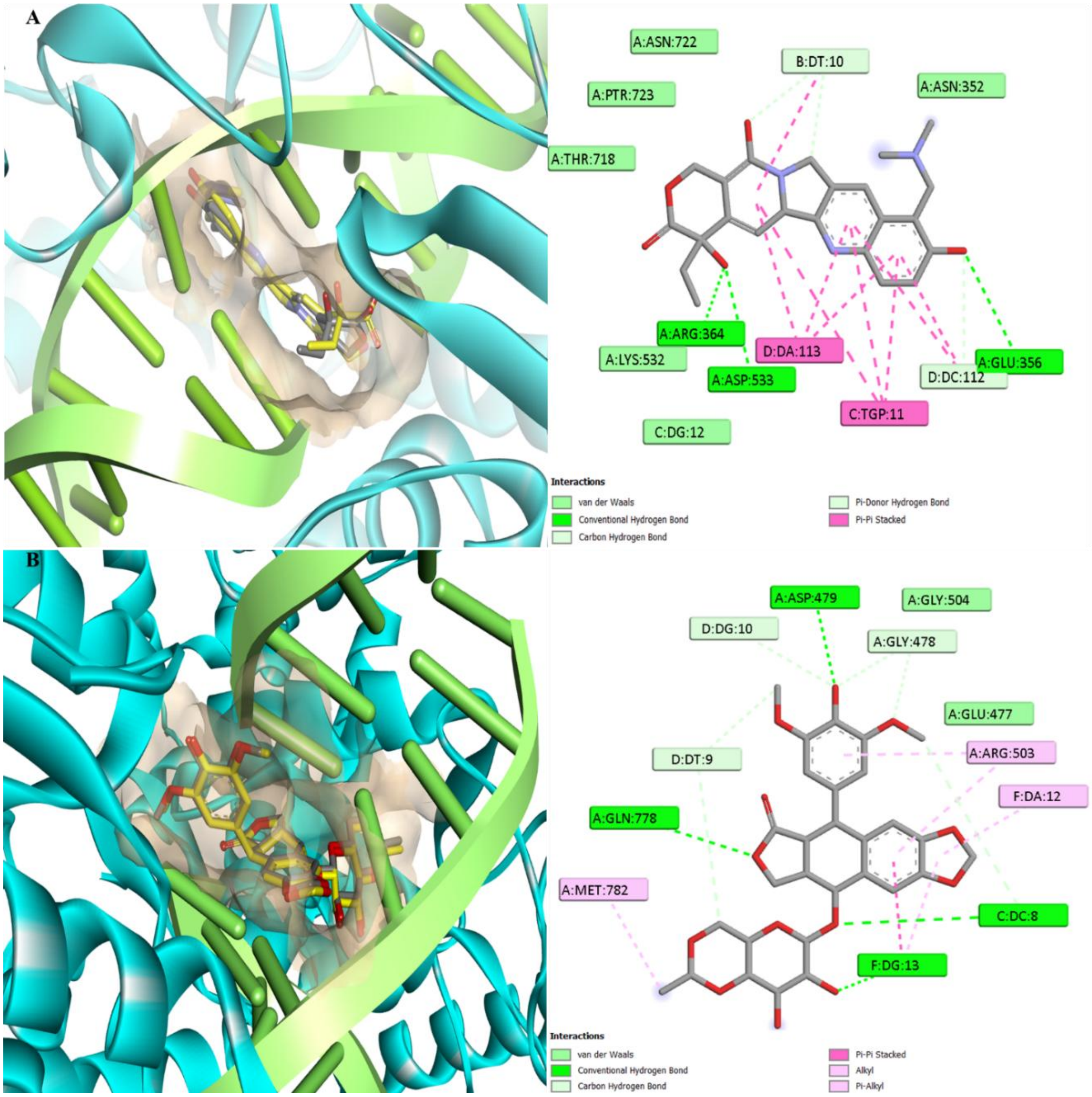

Figure S35. Overlay of the re-docking pose and the crystallized ligands "Yellow" inside the active site of (A)TOPI (PDB: 1K4T) with RMSD 0.760 and (B) TOPII (PDB: 3QX3) with RMSD 0.350.

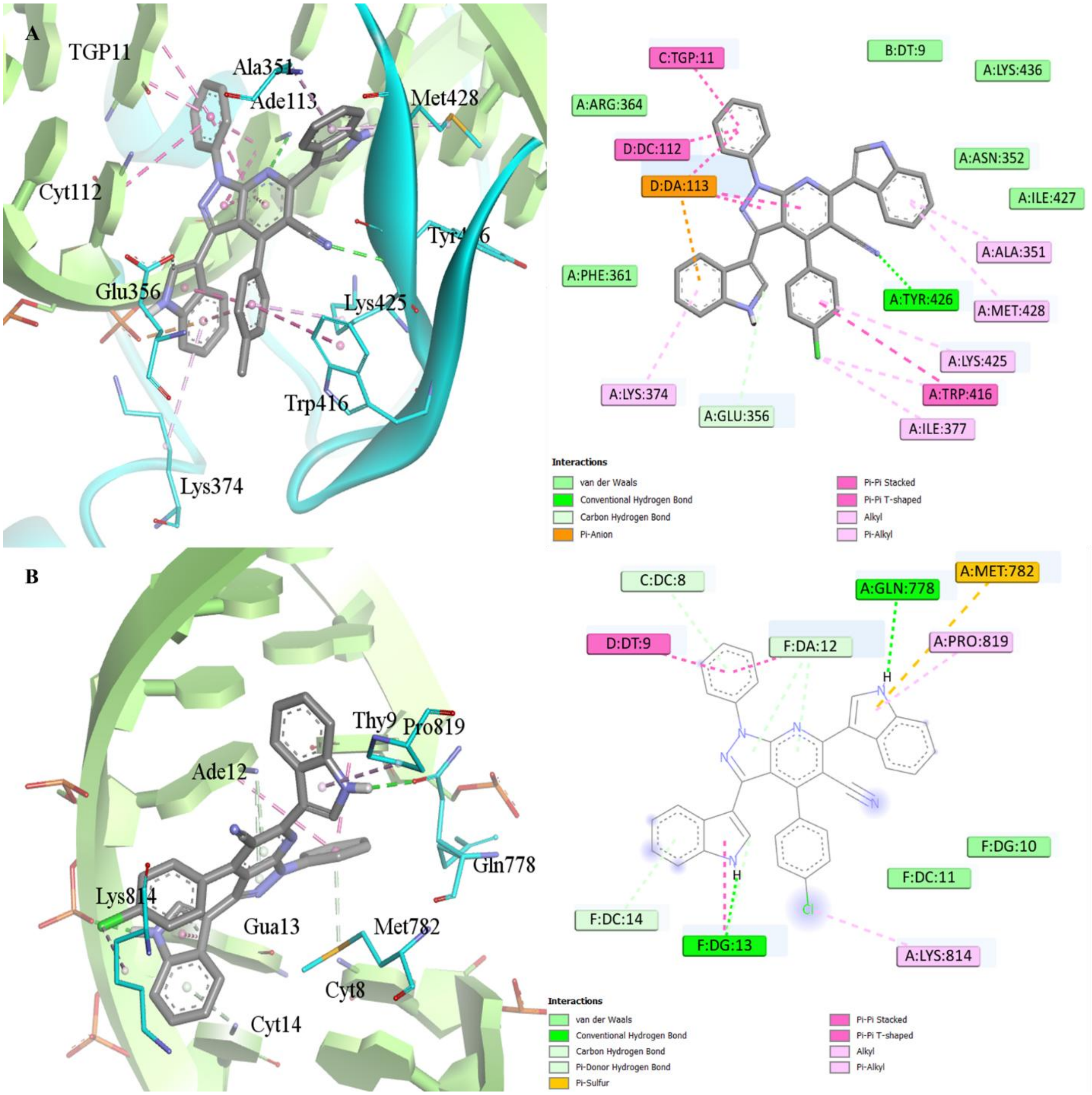

Figure 36. Molecular docking of **7e** inside the active site of (A)TOPI (PDB: 1K4T) with docking score -11.2 kcal/mol and (B) TOPII (PDB: 3QX3) with docking score -11.0 kcal/mol.
